# Type 2 innate immunity promotes the development of pulmonary fibrosis in Hermansky-Pudlak syndrome

**DOI:** 10.1172/jci.insight.178381

**Published:** 2024-11-22

**Authors:** Parand Sorkhdini, Kiran Klubock-Shukla, Selena Sheth, Dongqin Yang, Alina Xiaoyu Yang, Carmelissa Norbrun, Wendy J. Introne, Bernadette R. Gochuico, Yang Zhou

**Affiliations:** 1 Department of Molecular Microbiology and Immunology, Brown University, Providence, Rhode Island, USA.; 2 Medical Genetics Branch, National Human Genome Research Institute (NHGRI), NIH, Bethesda, Maryland, USA.

**Keywords:** Immunology, Pulmonology, Fibrosis, Genetic diseases, Innate immunity

## Abstract

Hermansky-Pudlak syndrome (HPS), particularly types 1 and 4, is characterized by progressive pulmonary fibrosis, a major cause of morbidity and mortality. However, the precise mechanisms driving pulmonary fibrosis in HPS are not fully elucidated. Our previous studies suggested that CHI3L1-driven fibroproliferation may be a notable factor in HPS-associated fibrosis. This study aimed to explore the role of CHI3L1-CRTH2 interaction on type 2 innate lymphoid cells (ILC2s) and explored the potential contribution of ILC2-fibroblast crosstalk in the development of pulmonary fibrosis in HPS. We identified ILC2s in lung tissues from patients with idiopathic pulmonary fibrosis and HPS. Using bleomycin-challenged WT and *Hps1*^–/–^ mice, we observed that ILC2s were recruited and appeared to contribute to fibrosis development in the *Hps1*^–/–^ mice, with CRTH2 playing a notable role in ILC2 accumulation. We sorted ILC2s, profiled fibrosis-related genes and mediators, and conducted coculture experiments with primary lung ILC2s and fibroblasts. Our findings suggest that ILC2s may directly stimulate the proliferation and differentiation of primary lung fibroblasts partially through amphiregulin-EGFR–dependent mechanisms. Additionally, specific overexpression of CHI3L1 in the ILC2 population using the *IL-7R^cre^* driver, which was associated with increased fibroproliferation, indicates that ILC2-mediated, CRTH2-dependent mechanisms might contribute to optimal CHI3L1-induced fibroproliferative repair in HPS-associated pulmonary fibrosis.

## Abstract 2

Hermansky-Pudlak syndrome is a group of inherited autosomal recessive disorders. In HPS types 1 and 4, pulmonary fibrosis develops in the fourth decade of life and is the major cause of morbidity and mortality. However, the mechanisms that mediate the development of pulmonary fibrosis in HPS have not been completely elucidated. Our previous studies demonstrate that exaggerated CHI3L1-driven fibroproliferation plays an important role in HPS fibrosis. In addition, the interaction between CHI3L1 and its receptor CRTH2, which occurs on various inflammatory cells including ILC2s, drives the fibroproliferative responses. The goal of the current research is to determine the effect of this CHI3L1-CRTH2 interaction on ILC2s and the contribution of ILC2-fibroblast crosstalk in the development of pulmonary fibrosis in HPS.

## Introduction

Hermansky-Pudlak syndrome (HPS) is a group of inherited autosomal recessive disorders that occur worldwide (1). Eleven genetic types (HPS-1 to HSP-11) have been described, with each mutation affecting the trafficking of lysosome-related organelles or vesicles. Due to a genetic founder effect, HPS-1 is particularly common in northwest Puerto Rico, where 1:1800 people are affected and the carrier frequency is 1 in 21 persons (2–8). Pulmonary fibrosis has been appreciated predominantly in HPS-1 and HPS-4 patients, whose genetic defects are in the biogenesis of lysosome-related organelle complex 3 (BLOC-3) (3, 9–12). Recently, BLOC-3 was identified as a guanine nucleotide exchange factor for the Rab small GTPase family member Rab32/38 (13). Correspondingly, HPS-associated lung disease occurs due to loss of BLOC-3–mediated Rab32/38 activity and defective intracellular protein trafficking. Due to the progressive nature of pulmonary fibrosis, it is the leading cause of death in HPS (14). However, although it is known that murine genetic models of HPS-1 manifest exaggerated injury and fibroproliferative repair responses to fibrogenic agents like bleomycin, the mechanism(s) by which defective protein trafficking leads to injury and fibrosis have not been adequately defined (15).

Our laboratory has published evidence that chitinase-like proteins play an important role in regulating the injury and repair responses in fibrotic lung diseases associated with HPS (16, 17). Chitinase 3–like 1 (CHI3L1) is the prototypic chitinase-like protein in the 18 glycosyl hydrolase gene family that binds, but does not degrade, chitin polysaccharides (16, 18–28). In patients with HPS pulmonary fibrosis (HPSPF) and idiopathic pulmonary fibrosis (IPF), high serum and lung levels of CHI3L1 are associated with disease progression and with alternatively activated circulating monocytes and macrophages, independent of its ability to bind and degrade chitin (16, 29). The *Hps1^−/−^* mouse has a null mutation of the *Hps1* gene and is the human homolog of HPS-1 (15, 30). Using this murine model, we demonstrated that a defect in CHI3L1 inhibition of epithelial apoptosis and exaggerated CHI3L1-driven fibroproliferation play important roles in HPSPF (16, 17). These studies demonstrate that abnormal IL-13Rα2 trafficking abrogates the antiapoptotic effects of CHI3L1 and contributes to the enhanced injury responses and sensitivity to apoptosis in BLOC-3 HPS patients and murine models of these disorders (16, 17). In contrast, CHI3L1 drives fibrosis via interactions with CRTH2, which traffics normally in BLOC-3 HPS patients and *Hps1*-deficient mice. These previous findings from our lab support the notion that CRTH2 is a receptor that interacts with CHI3L1, and a substantial role of the CHI3L1/CRTH2 axis in fibroproliferative responses in both the bleomycin model and HPSPF (17). However, the mechanisms and cell types through which CHI3L1 drives fibrotic repair and the manner in which these mechanisms are dysregulated in HPS have not been fully understood.

CRTH2 is a G protein–coupled receptor expressed by multiple immune cells and is commonly used to identify type 2 innate lymphoid cells (ILC2s) in the lung (31–33). ILCs are a family of innate immune cells that mirror the phenotypes and functions of T cells in adaptive immunity (34–36). Various studies have reported that IL-13–expressing ILC2s play a key role in antihelminth innate immunity, asthma, and other allergic diseases (31–33, 37–40). Recently, studies have implicated BM-derived ILC2s in the pathogenesis of pulmonary fibrosis in both mice and humans. ILC2s are recruited through the IL-33/ST2 pathway and contributed to fibroblast activation to promote lung fibrosis (41–43). The fact that CHI3L1 mediates its profibrotic effects through CRTH2, and that CRTH2-positive ILC2s are indicated in pulmonary fibrosis development in both humans and mice, allows us to speculate that CRTH2, via its expression on ILC2s, mediates the cellular and tissue effects of CHI3L1 that lead to fibroproliferation (41–43).

We hypothesize that ILC2s are recruited to the lungs of HPS patients and *Hps1*^−/−^ mice via the CHI3L1/CRTH2 pathway, where they stimulate fibroblast proliferation and differentiation and contribute to the development of HPSPF. The goals of the current research are to determine the effect of CHI3L1-CRTH2 interaction on ILC2s and the contribution of ILC2 activation in pulmonary fibrosis in the *Hps1*^−/−^ mouse model. Our studies demonstrated that ILC2s were recruited to the lungs of IPF and HPSPF patients. The *Hps1*^−/−^ mice developed exacerbated lung fibrosis when challenged with bleomycin compared with WT mice. CHI3L1 interacted with CRTH2 to augment fibrotic responses because collagen accumulation was notably upregulated when CHI3L1 was overexpressed and was reduced in the mice treated with a CRTH2 inhibitor. ILC2s were recruited and contributed to fibrosis development in the *Hps1*^−/−^ mice, and CRTH2 played a substantial role in ILC2 accumulation. ILC2s sorted from bleomycin-challenged *Hps1*^−/−^ mice had distinct profibrotic gene expression profiles and expressed elevated levels of IL-5, IL-13, and amphiregulin (AREG). They directly stimulated the proliferation and differentiation of primary lung fibroblasts partially through AREG-EGFR–dependent mechanisms. In addition, overexpression of CHI3L1 specifically in the ILC2 population using the *IL-7R^cre^* driver was sufficient to induce increased fibroproliferation. Overall, our findings demonstrate that an ILC2-mediated, CRTH2-dependent mechanism(s) contributes to optimal CHI3L1-induced fibroproliferative repair in HPSPF.

## Results

### ILC2s are increased in human HPSPF and localize to fibrotic regions of the lung.

**Figure 1:**
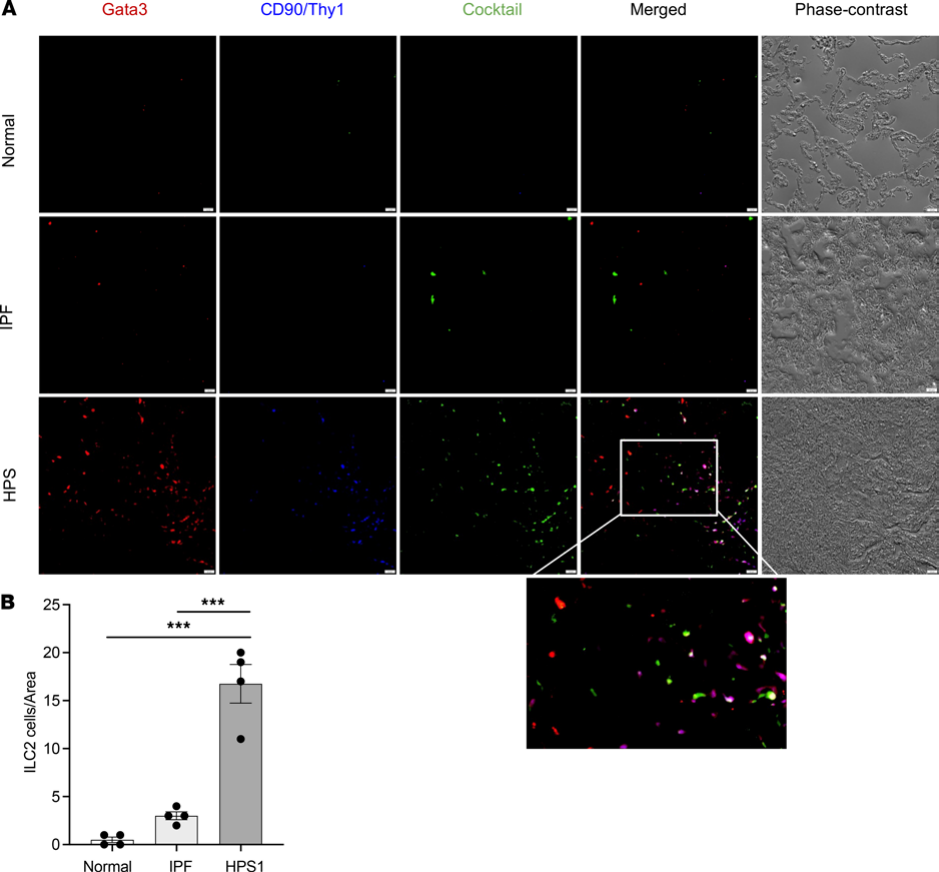
ILC2s are increased in human HPS lungs. (**A**) ILC2 staining in human lung tissues. Anti-GATA3, -CD90/Thy1, and a cocktail of 4 antibodies against CD3, CD56, CD20, and CD79α were employed to stain human lung tissues in normal individuals, idiopathic pulmonary fibrosis (IPF) patients, and HPS patients. ILC2s are identified as GATA3^+^CD90/Thy1^+^, and antibody cocktail–negative cells (purple cells). The corresponding phase-contrast image shows lung tissue architecture in normal individuals, and pathologic changes in IPF and HPS lungs. (**B**) Counting of ILC2s (normalized per area) under ×40 magnification using an immunofluorescence microscope. Values are mean ± SEM with a minimum of 4 samples in each group. Comparisons between groups were conducted by 2-way ANOVA with Bonferroni’s post hoc test. ***P ≤ 0.001. Scale bars: 20 μm.

To visualize the recruitment and localization of ILC2s in formalin-fixed, paraffin-embedded (FFPE) human lung tissues, immunofluorescent staining was performed in samples from normal individuals, IPF, and HPSPF patients. ILC2s are characterized by the expression of GATA-binding protein 3 (GATA3) and CD90. GATA3 and CD90 staining was employed as a positive identifier of ILC2s in combination with a cocktail of 4 lymphoid lineage marker antibodies to exclude non-ILCs: CD3 (T cells), CD20 and CD79α (B cells/plasma cells), and CD56 (NK cells) (44). ILC2s are identified as GATA3^+^CD90^+^Lineage^–^ cells (purple color), where Th2 cells and other immune populations are identified in red, green, or white (Figure 1A). Quantitative analysis of ILC2s in all 3 groups was achieved using ImageJ software (NIH, https://imagej.net/) (Figure 1B). Our study demonstrated that the number of ILC2s was significantly increased in HPSPF patients compared with IPF patients and normal individuals, as determined using immunofluorescence. DAPI staining was performed to visualize GATA3-positive and lineage cocktail–negative cells (Supplemental Figure 1A; supplemental material available online with this article; https://doi.org/10.1172/JCI17838DS1). To investigate localization of these cells in the fibrotic regions, α-smooth muscle actin (α-SMA) staining was employed to identify fibrotic foci (cascade blue, Supplemental Figure 1B) (45, 46). We found a significant increase in the recruitment of GATA3-positive and lineage cocktail–negative cells to the fibrotic regions of IPF and HPS-1 lungs (Supplemental Figure 1B). These findings demonstrate the substantial recruitment of ILC2 cells to the fibrotic regions as a key event in the development of profibrotic conditions in lungs of IPF and HPS-1 patients.

### ILC2s are increased in HPS/bleomycin-induced lung fibrosis and are recruited via chemoattractant receptor–homologous molecules expressed on Th2 cells (CRTH2).

**Figure 2:**
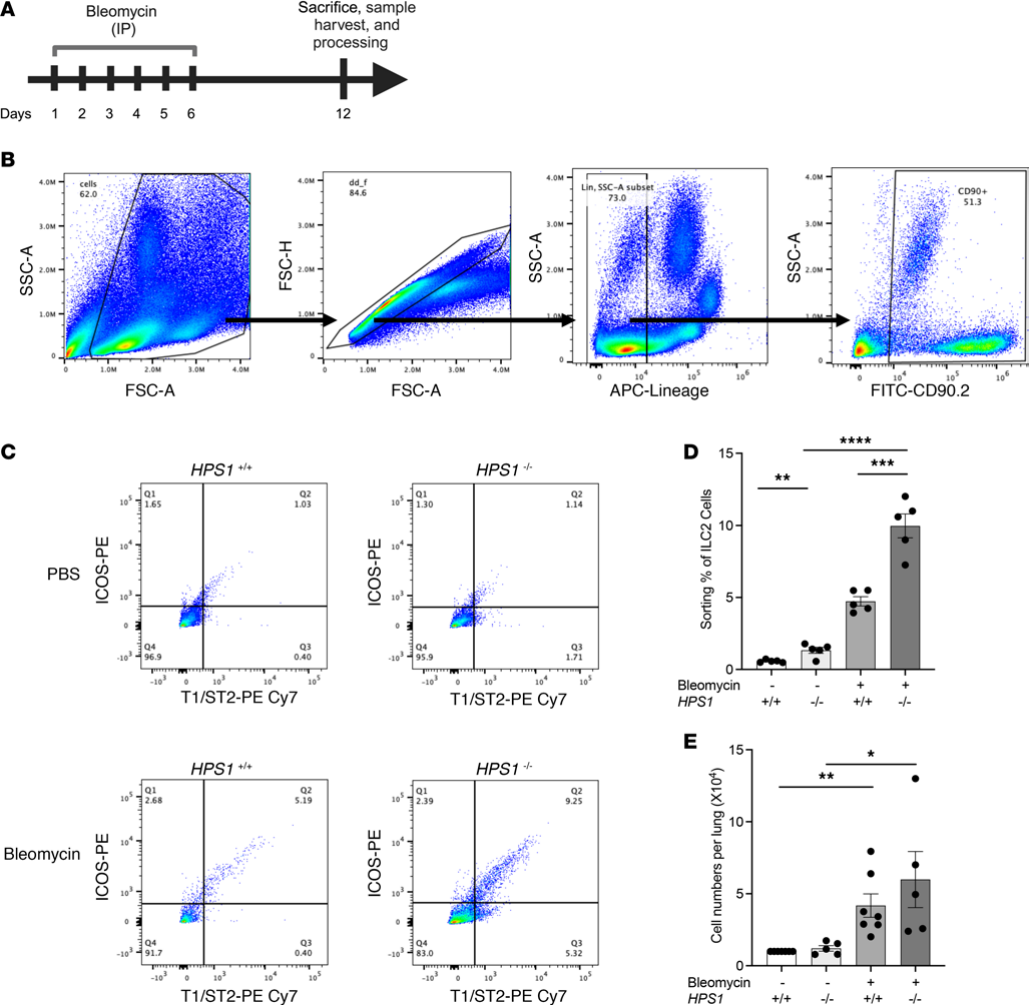
ILC2s are increased in HPS/bleomycin-induced lung fibrosis. WT and *HPS1^–/–^* mice were subjected to i.p. bleomycin administration, primary ILC2s were sorted from mouse lungs, and ILC2 numbers were assessed by flow cytometry. (**A**) Schematic of the experiment. (**B**) Gating strategy of CD90-positive, Lineage-negative, T1/ST2-positive, and ICOS-positive ILC2s. (**C** and **D**) Representative charts and quantification of increased percentages of pulmonary ILC2s in the lungs of bleomycin-treated *HPS1^–/–^* mice. (**E**) Increased pulmonary ILC2 numbers in the lungs of bleomycin-treated *HPS1^–/–^* mice. Values are mean ± SEM with a minimum of 8 mice in each group. Comparisons between groups were conducted by 2-way ANOVA with Bonferroni’s post hoc test. *P ≤ 0.05; **P ≤ 0.01; ***P ≤ 0.001; ****P ≤ 0.0001.

**Figure 3:**
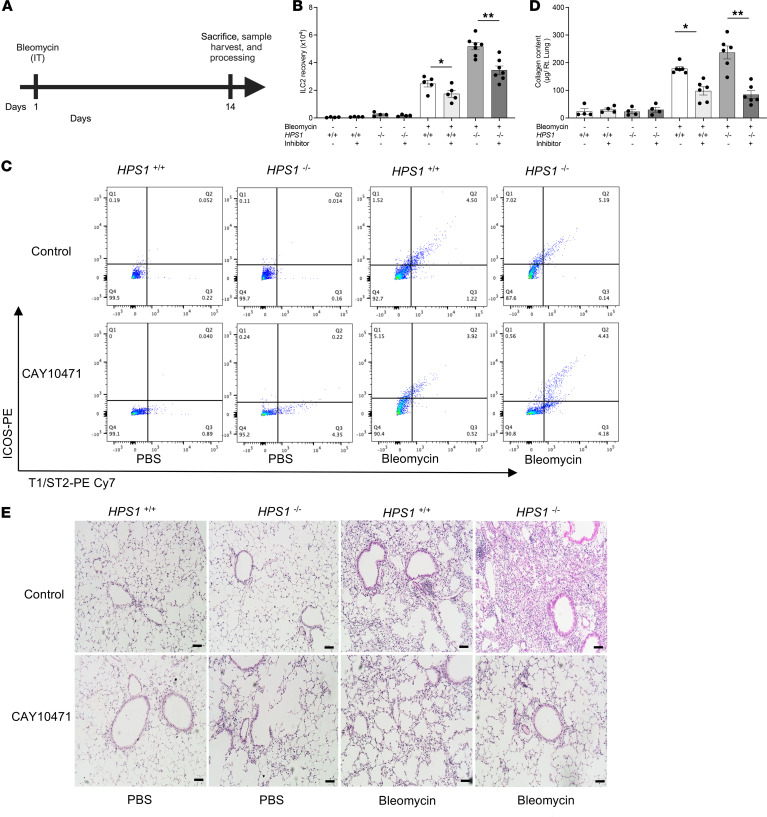
CRTH2 inhibition prevents ILC2 recruitment and fibrosis development in HPS/bleomycin-induced lung fibrosis. (**A**) Schematic of the experiment. WT and *HPS1^–/–^* mice were subjected to a single dose of intratracheal (i.t.) bleomycin administration. (**B**–**E**) Mice were treated with CRTH2 inhibitor or its vehicle control. (**B**) ILC2 number assessed by flow cytometry. (**C**) Gating strategy of CD90-positive, Lineage-negative, T1/ST2-positive, and ICOS-positive ILC2s. (**D**) A Sircol assay was used to assess the levels of collagen accumulation in the lung. (**E**) Representative histopathology of lung sections stained with H&E to depict the degree of fibrosis. Scale bars: 200 μm. *P ≤ 0.05; **P ≤ 0.01 by 2-way ANOVA with Bonferroni’s post hoc test.

**Figure 4:**
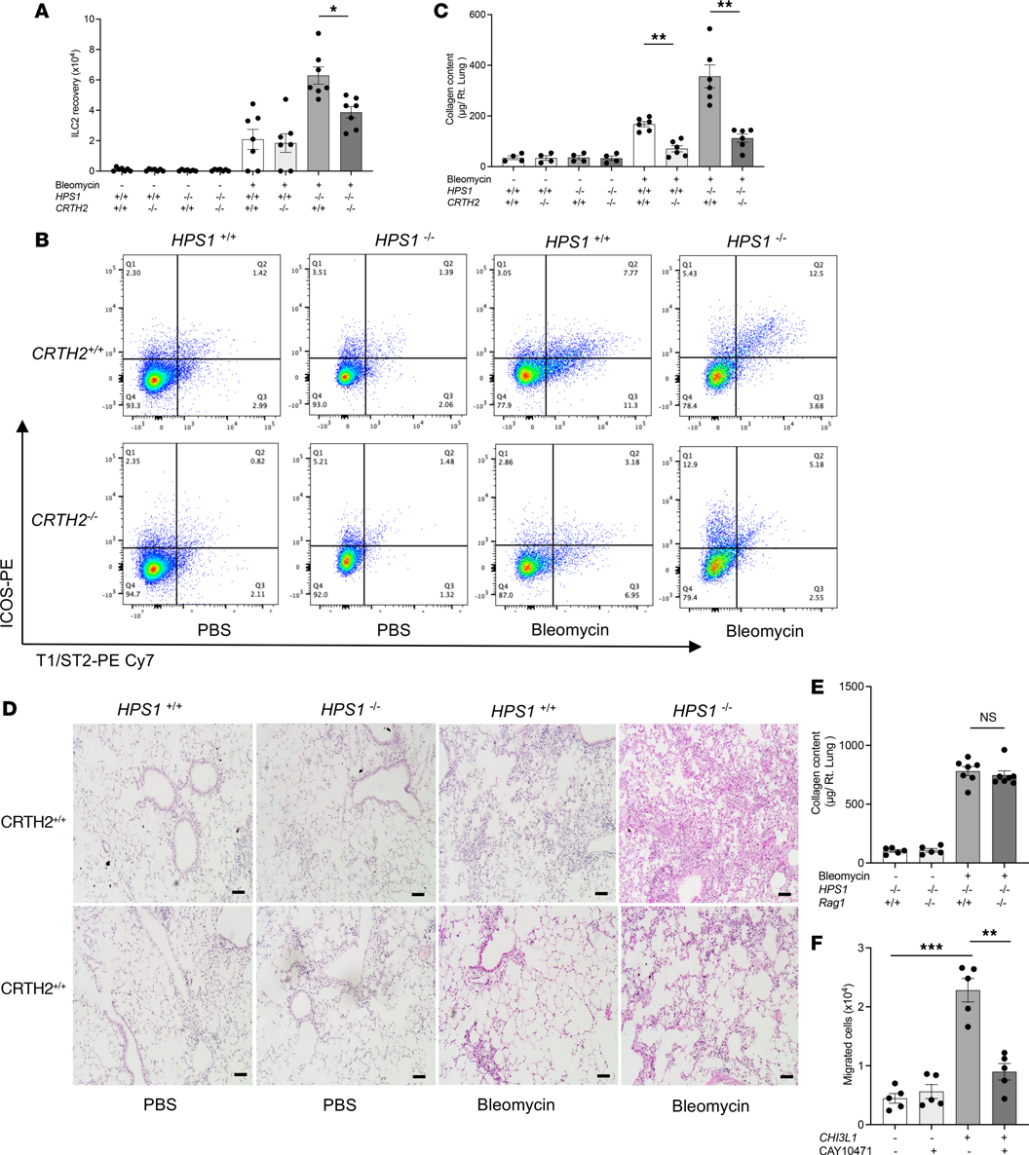
ILC2s are recruited via CHI3L1-CRTH2–dependent mechanisms in HPS/bleomycin-induced lung fibrosis. WT, CRTH2-knockout (*CRTH2^–/–^*), *HPS1^–/–^*, and *HPS1^–/–^* *CRTH2^–/–^* double-mutant mice were subjected to intratracheal (i.t.) bleomycin administration. (**A**) Lung ILC2 number was assessed by flow cytometry. (**B**) Gating strategy of CD90-positive, Lineage-negative, T1/ST2-positive, and ICOS-positive ILC2s. (**C**) Sircol assay was used to assess the levels of collagen accumulation in the lung. (**D**) Representative histopathology of lung sections stained with H&E to depict the degree of fibrosis. (**E**) *HPS1^–/–^* *Rag1^–/–^* mice were subjected to a single dose of i.t. bleomycin administration. A Sircol assay was used to assess the levels of collagen accumulation in the lung. (**F**) An ILC2 recruitment assay was performed in Transwell plates in vitro. Values are mean ± SEM with a minimum of 8 mice in each group. Comparisons between groups were conducted by 2-way ANOVA with Bonferroni’s post hoc test. *P ≤ 0.05; **P ≤ 0.01; ***P ≤ 0.001. Scale bars: 200 μm.

We next sought to determine whether ILC2s are recruited in mouse models of HPS-associated lung fibrosis. WT and *Hps1*^−/−^ mice were subjected to intraperitoneal (i.p.) bleomycin administration to induce lung fibrosis (Figure 2A), mouse lungs were perfused with PBS to remove circulating cells, and frequency of Lineage^–^Thy1.2^+^ICOS^+^T1/ST2^+^ ILC2s was quantitated at baseline and after bleomycin challenge using flow cytometry (Figure 2B and Supplemental Figure 2). We observed increased percentages of pulmonary ILC2s in the lungs of bleomycin-treated *Hps1*^−/−^ mice (Figure 2, C and D). Consistent with these results, we observed increased pulmonary ILC2 number in the lungs of bleomycin-treated *Hps1*^−/−^ mice (Figure 2E). Our previous studies showed that CRTH2 plays an important role in the augmented fibrotic responses in the bleomycin-induced pulmonary fibrosis in *Hps1*^−/−^ mice, as collagen accumulation was significantly upregulated when CHI3L1 was overexpressed and was reduced in the mice treated with a CRTH2 inhibitor (16, 17). Based on our findings that *Hps1*^−/−^ mice had a significantly greater ILC2 population in the lung compared with the control group and that CRTH2 is widely expressed on ILC2s, we hypothesized that CRTH2 played a role in ILC2 accumulation in the bleomycin-induced pulmonary fibrosis. WT and *Hps1*^−/−^ mice were treated with a CRTH2 inhibitor (i.e., CAY10471) after bleomycin challenge. We previously found that *Hps1*^−/−^ mice treated with the CRTH2 inhibitor were protected from pulmonary fibrosis (16, 17). In the current study (Figure 3A), we found that ILC2 accumulation was significantly blocked in mice that were treated with the CRTH2 inhibitor (Figure 3, B and C). Consistent with previous findings, bleomycin-induced fibrosis was also diminished in mice that were treated with the CRTH2 inhibitor (Figure 3, D and E). To confirm the role of CRTH2 in ILC2 accumulation in the lungs, *Hps1*^−/−^ *CRTH2*^−/−^ double-mutant mice were generated and subjected to bleomycin administration. We found that lung accumulation of ILC2s was decreased in bleomycin-challenged *Hps1*^−/−^ *CRTH2*^−/−^ double-mutant mice compared with *Hps1*^−/−^ mice (Figure 4, A and B). Consistently, bleomycin-induced fibrosis was also diminished in *Hps1*^−/−^ *CRTH2*^−/−^ double-mutant mice compared with *Hps1*^−/−^ mice (Figure 4, C and D). In contrast, when *Hps1*^−/−^ mice were bred with *Rag1*^−/−^ mice to deplete T cells, *Hps1*^−/−^ *Rag1*^−/−^ double-mutant mice were not protected from bleomycin-induced lung fibrosis development (Figure 4E), suggesting Th2s did not contribute to fibrosis development in this model. In vitro, ILC2s were recruited to CHI3L1-containing media, and the recruitment was blocked by the CRTH2 inhibitor (Figure 4F). Based on our findings, CRTH2 plays a key role in ILC2 accumulation in the bleomycin-induced pulmonary fibrosis in *Hps1*^−/−^ mice.

### ILC2s contribute to HPS/bleomycin-induced lung fibrosis.

**Figure 5:**
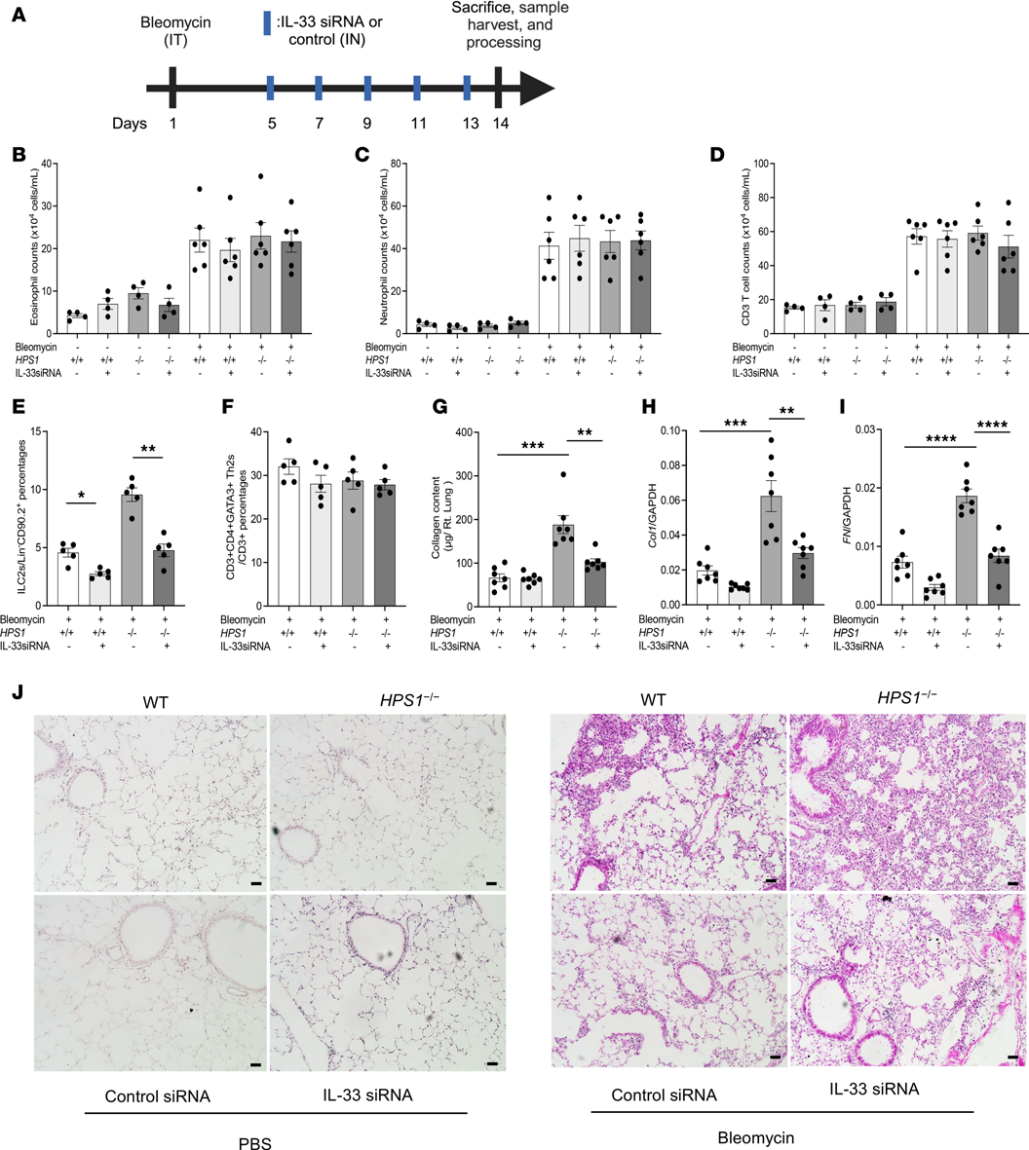
IL-33–mediated ILC2 activation contributes to HPS/bleomycin-induced lung fibrosis. WT and *HPS1^–/–^* mice were subjected to intratracheal (i.t.) bleomycin administration. Mice were treated with IL-33 siRNA (every other day, 3 nmol/mouse) or its scrambled control. (**A**) Schematic of the experiment. (**B**–**D**) Numbers of BAL eosinophils, neutrophils, and T cells were not altered by IL-33 siRNA treatment. (**E** and **F**) Percentages of lung ILC2s and Th2s were assessed by flow cytometry. (**G**) Total lung collagen was quantified using a Sircol assay on day 14. (**H** and **I**) Gene expression of COL-A1 mRNA and fibronectin mRNA was measured by RT-PCR. (**J**) Representative histopathology of lung sections stained with H&E to depict the degree of fibrosis. Values are mean ± SEM with 7–9 mice in each group. Comparisons between groups were conducted by 2-way ANOVA with Bonferroni’s post hoc test. *P ≤ 0.05; **P ≤ 0.01; ***P ≤ 0.001; ****P ≤ 0.0001. Scale bars: 100 μm.

We next sought to determine the role of ILC2s in fibrotic tissue repair responses in the bleomycin-induced pulmonary fibrosis in *Hps1*^−/−^ mice. IL-33 is a major cytokine that is secreted by lung epithelial cells and activates ILC2s (47, 48). Thus, we used IL-33 siRNA to knock down the expression of IL-33 and ILC2 activation in vivo (Figure 5A). We found that IL-33 siRNA did not alter the recruitment of eosinophils, neutrophils, and T cells in the lung (Figure 5, B–D). It significantly diminished the percentages of ILC2s, while it had no effect on the percentages of Th2s (Figure 5, E and F). Importantly, compared with scrambled siRNA–treated mice, the exaggerated collagen accumulation, α1 procollagen, and fibronectin expression in *Hps1*^−/−^ mice were significantly decreased by intranasal IL-33 siRNA treatment (Figure 5, G–I). Consistent histological findings were observed in that IL-33 siRNA treatment diminished fibrosis development in *Hps1*^−/−^ mice (Figure 5J). To test whether ILC2 deficiency in *Hps1*^−/−^ mice also affects bleomycin-induced pulmonary fibrosis development, *Rora^fl/fl^* *IL-7R^cre^* mice, in which ILC2s are depleted (49–51), were bred with *Hps1*^−/−^ mice to examine the specific roles of ILC2s in an i.p.-bleomycin model (Supplemental Figure 3A). ILC2 depletion did not alter the recruitment of eosinophils, neutrophils, and T cells in the lung (Supplemental Figure 3, B–D). It significantly diminished the percentages of ILC2s, while it had no effect on the percentages of Th2s (Supplemental Figure 3, E and F). Our result demonstrated that bleomycin-induced collagen accumulation was significantly diminished in *Hps1*^−/−^ *Rora^fl/fl^* *IL-7R^cre^* mice (Supplemental Figure 3G). Consistently, α1 procollagen and fibronectin expression, and the development of lung fibrosis were significantly decreased in *Hps1*^−/−^ *Rora^fl/fl^* *IL-7R^cre^* mice compared with *Hps1*^−/−^ mice (Supplemental Figure 3, H–J). Taken together, these results show that ILC2s were increased and contributed to fibrosis development in the *Hps1*^−/−^ mice, and CRTH2 played a noteworthy role in ILC2 accumulation. Our findings demonstrate that ILC2-mediated, CRTH2-dependent mechanism(s) contributed to optimal CHI3L1-induced amplified fibrotic responses in the bleomycin-induced pulmonary fibrosis in *Hps1*^−/−^ mice.

In addition to ILC2s, we documented the accumulation of eosinophils and macrophages that may also be involved in type 2 immunity. We found that bronchoalveolar lavage (BAL) eosinophilia and mRNA levels of eotaxin were not altered in *Hps1*^−/−^ mice (Supplemental Figure 4, A and B). Interestingly, alveolar macrophages appeared to have been skewed toward a “profibrotic” phenotype by expressing more CD206 (Supplemental Figure 4, C and D). Surprisingly, while macrophage depletion significantly blocked fibrosis development in WT animals, it did not alter the fibroproliferative response in *Hps1*^−/−^ mice (Supplemental Figure 4E). These data suggest that the profibrotic role of ILC2s is specific to the HPS model, and macrophages do not directly contribute to fibrosis development in *Hps1*^−/−^ mice. In addition, we did not see any differences in IL-13 production in the 2 groups (Supplemental Figure 4F), and IL-13–blocking antibody did not prevent the development of fibrosis in this model (Supplemental Figure 4G). On the contrary, when challenged with house dust mite (HDM) extract to induce typical type 2 immune responses, *Hps1*^−/−^ mice had increased numbers of eosinophils as well as eotaxin mRNA levels in the lung (Supplemental Figure 4, H and I).

### ILC2s sorted from bleomycin-challenged Hps1^−/−^ mice have distinct profibrotic gene expression profiles.

**Figure 6:**
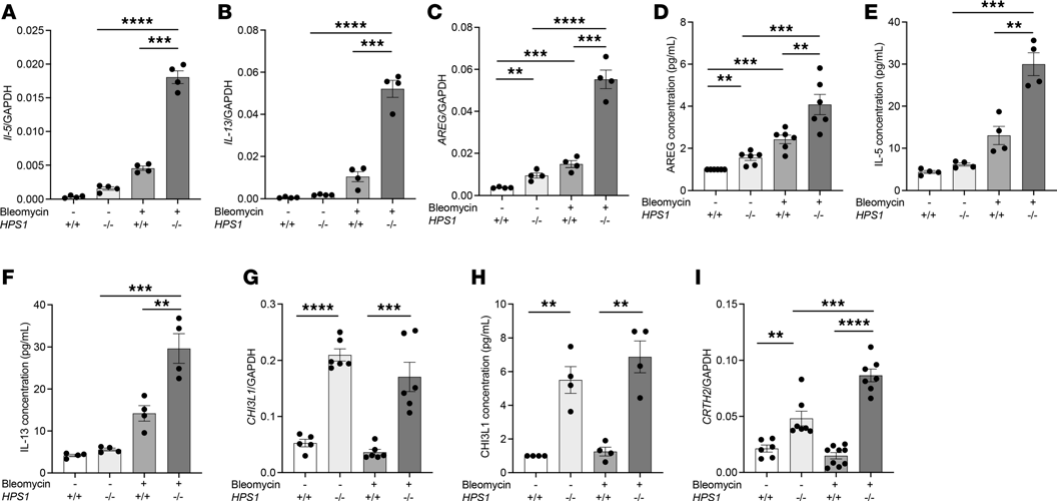
ILC2s sorted from bleomycin-challenged *HPS1^–/–^* mice express high levels of IL-5, IL-13, amphiregulin (AREG), CRTH2, and CHI3L1. (**A**–**C**, **G**, and **I**) Gene expression levels for IL-5, IL-13, AREG, and CRTH2. (**D**–**F** and **H**) Soluble IL-5, IL-13, AREG, and CHI3L1 produced by ILC2s were measured in the culture supernatants of ILC2s sorted from WT and *HPS1^–/–^* mice. Values are mean ± SEM with 7–9 mice in each group. Comparisons between groups were conducted by 2-way ANOVA with Bonferroni’s post hoc test. *P ≤ 0.05; **P ≤ 0.01; ***P ≤ 0.001; ****P ≤ 0.0001.

ILC2s release type 2 cytokines (such as IL-5 and IL-13) and growth factors (such as AREG) in inflamed and injured lung tissues in patients with chronic obstructive pulmonary disease and asthma. These type 2 cytokines and growth factors can contribute to the development of lung fibrosis by inducing production of extracellular matrix proteins and myofibroblast differentiation. Therefore, we next sought to determine whether IL-5, IL-13, and AREG produced by ILC2s were altered in *Hps1*^−/−^-associated lung fibrosis. Our gene expression analysis using real-time PCR showed significantly increased expression of IL-5, IL-13, and AREG from sorted ILC2s in bleomycin-challenged *Hps1*^−/−^ mice (Figure 6, A–C). Consistently, the concentration of AREG released in ILC2 culture supernatant followed a similar pattern and increased significantly in bleomycin-challenged *Hps1*^−/−^ mice compared with WT controls (Figure 6D). Moreover, IL-5 and IL-13 concentrations in cell culture supernatant were significantly elevated in *Hps1*^−/−^ cells compared with WT controls (Figure 6, E and F). We also examined the expression of CHI3L1 and its receptor CRTH2 in sorted ILC2s as major contributors to the augmented fibroproliferative repair in *Hps1*^−/−^ mice. Interestingly, our data demonstrated that, at baseline, CRTH2 and CHI3L1 mRNA expression levels in sorted ILC2s and CHI3L1 concentrations in cell culture supernatant were significantly elevated in *Hps1*^−/−^ mice compared with WT controls (Figure 6, G–I). In addition, ILC2s sorted from bleomycin-challenged *Hps1*^−/−^ mice had significantly higher expression of CRTH2 compared with PBS-treated *Hps1*^−/−^ mice (Figure 6I). These studies demonstrate that ILC2s that regulate fibrosis development in bleomycin-challenged *Hps1*^−/−^ mice are characterized by high expression of IL-5, IL-13, AREG, CRTH2, and CHI3L1.

**Figure 7:**
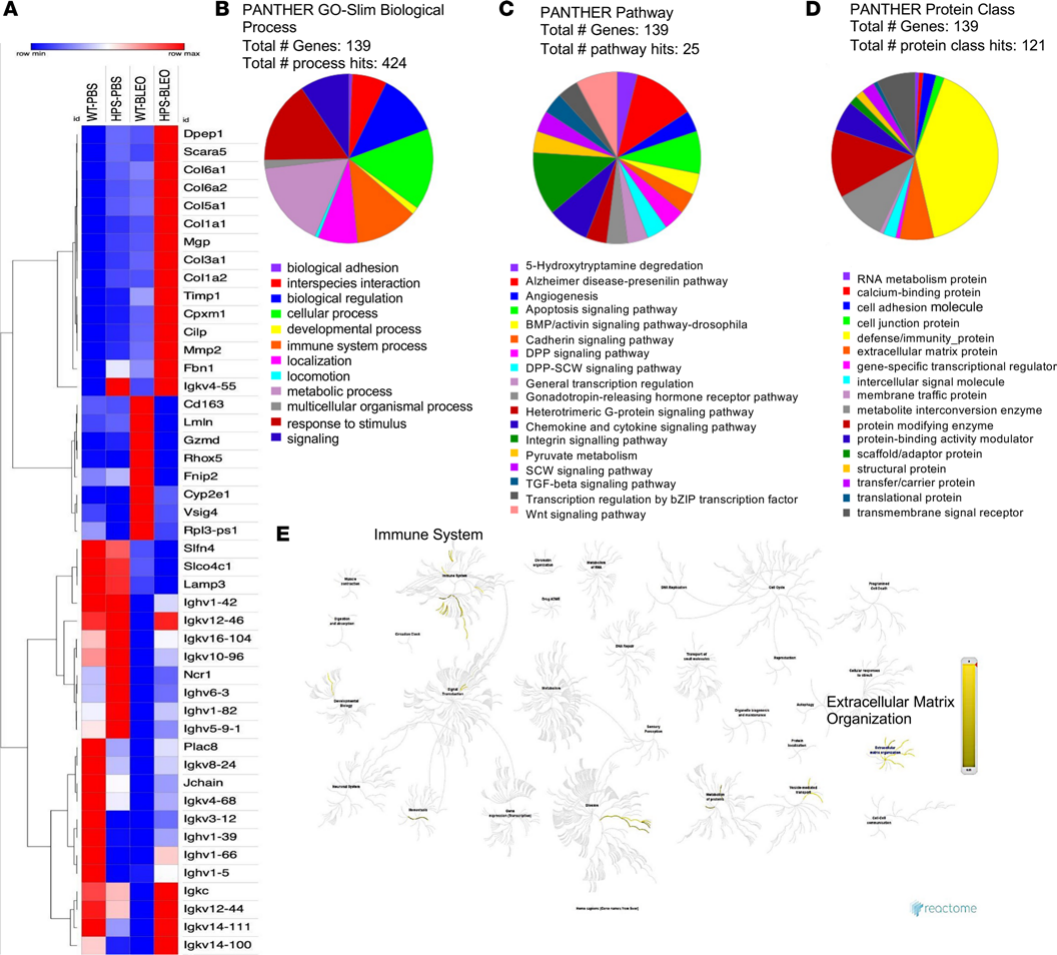
ILC2s sorted from bleomycin-challenged *HPS1^–/–^* mice exhibited a unique profibrotic gene expression profile. (**A**) Gene expression heatmap analyzed by Python. There are 46 genes that were differentially expressed. In the upper cluster, upregulated genes are visible. Many of them play a role in a variety of matrix remodeling and collagen production in various lung diseases and cancers. (**B**–**D**) Three pie charts displaying the molecular function (MF), biological process, and protein class of top genes that were most altered in ILC2s isolated from *HPS1^–/–^* mice compared with WT mice upon bleomycin treatment. (**E**) Reactome analysis highlights the immune system and extracellular matrix organization pathways that were altered in ILC2s isolated from *HPS1^–/–^* mice compared with WT mice upon bleomycin treatment.

Using transcriptomic approaches, we profiled the expression of genes and soluble mediators that may contribute to fibrosis development from sorted ILC2s in WT and *Hps1^−/−^* mice challenged with bleomycin. Our RNA-Seq data showed upregulation of genes responsible for matrix remodeling and collagen production in sorted ILC2 cells from bleomycin-challenged *Hps1*^−/−^ mice. Scavenger receptor class A member 5 (*Scara5*), collagen type VI α2 chain (*Col6a2*), collagen type V α1 chain (*Col5a1*), collagen type I α1 chain (*Col1a1*), matrix Gla protein (*Mgp*), collagen type III α1 chain (*Col3a1*), collagen type I α2 chain (*Col1a2*), TIMP metallopeptidase inhibitor (*Timp1*), matrix metallopeptidase 2 (*Mmp2*), and fibrillin 1 (*Fbn1*) are some of upregulated genes depicted in the upper side cluster of the heatmap (Figure 7A). Further molecular function, biological process, and protein class analysis using PantherDB (https://www.pantherdb.org Accessed August 8, 2023.) revealed the top genes that were most altered upon bleomycin treatment in *Hps1^−/−^* mice compared with WT mice (2-fold change of log_2_-based, *P* < 0.05) (Figure 7, B–D). Reactome analysis (https://reactome.org Accessed August 8, 2023.) highlighted that immune pathways and extracellular matrix organization were altered in ILC2s isolated from *Hps1^−/−^* mice compared with WT mice upon bleomycin treatment (Figure 7E).

### ILC2s stimulate fibroblast proliferation and differentiation in in vitro coculture.

**Figure 8:**
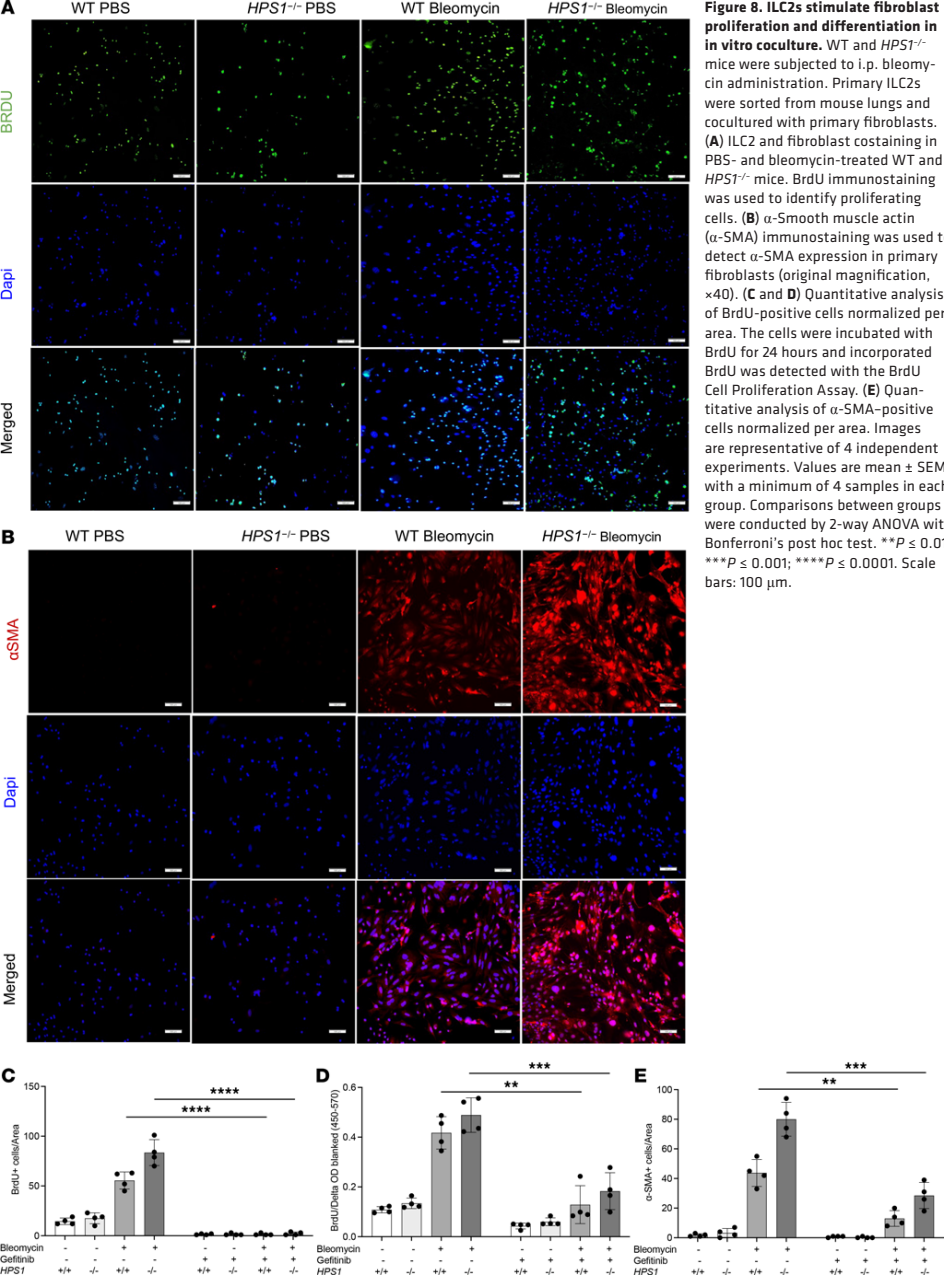
ILC2s stimulate fibroblast proliferation and differentiation in in vitro coculture. WT and *HPS1^–/–^* mice were subjected to i.p. bleomycin administration. Primary ILC2s were sorted from mouse lungs and cocultured with primary fibroblasts. (**A**) ILC2 and fibroblast costaining in PBS- and bleomycin-treated WT and *HPS1^–/–^* mice. BrdU immunostaining was used to identify proliferating cells. (**B**) α-Smooth muscle actin (α-SMA) immunostaining was used to detect α-SMA expression in primary fibroblasts (original magnification, ×40). (**C** and **D**) Quantitative analysis of BrdU-positive cells normalized per area. The cells were incubated with BrdU for 24 hours and incorporated BrdU was detected with the BrdU Cell Proliferation Assay. (**E**) Quantitative analysis of α-SMA–positive cells normalized per area. Images are representative of 4 independent experiments. Values are mean ± SEM with a minimum of 4 samples in each group. Comparisons between groups were conducted by 2-way ANOVA with Bonferroni’s post hoc test. **P ≤ 0.01; ***P ≤ 0.001; ****P ≤ 0.0001. Scale bars: 100 μm.

To investigate the contribution of ILC2-fibroblast crosstalk in the development of HPSPF, we performed coculture experiments with primary lung ILC2s and fibroblasts to determine the interactions between these 2 cell types. WT and *Hps1^−/−^* mice were subjected to i.p. bleomycin administration and primary ILC2s were sorted from mouse lung and cocultured with primary lung fibroblast. Subsequently, ILC2s and fibroblasts from PBS- and bleomycin-treated WT and *Hps1*^−/−^ mice were stained with bromodeoxyuridine (BrdU) to identify proliferating cells (Figure 8A) and α-SMA immunostaining to detect α-SMA^+^ myofibroblasts (Figure 8B). We found that ILC2s sorted from bleomycin-challenged WT mice significantly stimulated the proliferation and differentiation of primary lung fibroblasts. Moreover, ILC2s sorted from bleomycin-challenged *Hps1*^−/−^ mice were able to further stimulate the proliferation and differentiation of primary lung fibroblasts (Figure 8, A–E). To further consolidate the contribution of ILC2-fibroblast crosstalk in the development of HPSPF, gefitinib was added to the culture media to block AREG signaling. We found that gefitinib significantly reduced the ability of ILC2s to stimulate fibroblast proliferation and expression of α-SMA (Figure 8, C–E, and Supplemental Figure 5, A and B). We did not observe any cell proliferation or differentiation in ILC2-only or fibroblast-only cultures (Supplemental Figures 6–8). These studies demonstrate that ILC2s from bleomycin-challenged *Hps1*^−/−^ mice directly stimulated the proliferation and differentiation of primary lung fibroblasts partially through AREG-EGFR–dependent mechanisms.

### Overexpression of CHI3L1 in ILC2s leads to increased fibrosis development.

**Figure 9:**
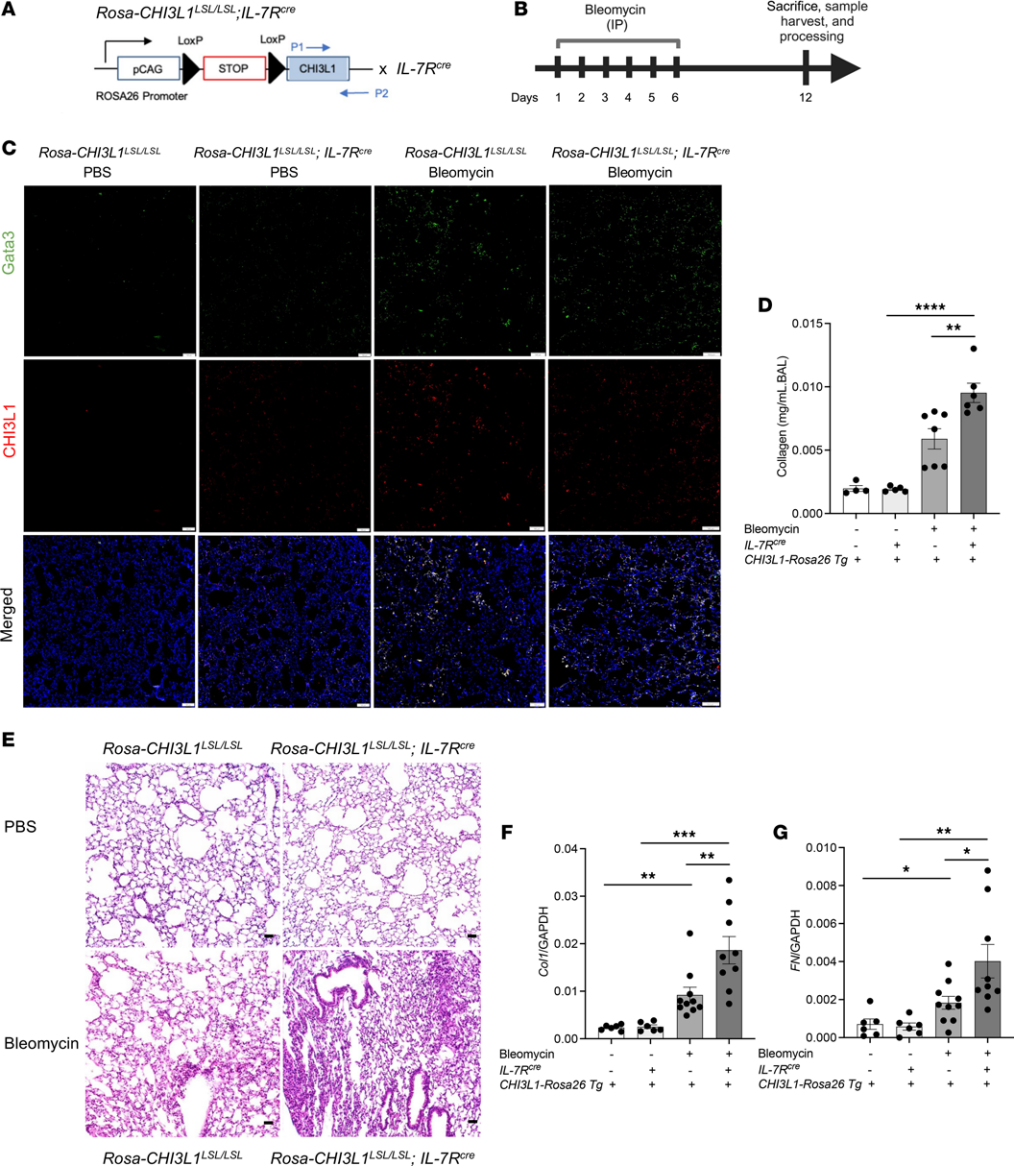
Overexpression of CHI3L1 in ILC2s leads to increased fibrosis development. (**A**) Schematic diagram for the generation of *Rosa-CHI3L1^LSL/LSL^* *IL-7R^cre^* transgenic mice. *Rosa-CHI3L1^LSL/LSL^* and *Rosa-CHI3L1^LSL/LSL^* *IL-7R^cre^* mice were subjected to i.p. bleomycin administration. (**B**) Schematic of the experiment. (**C**) CHI3L1 and GATA3 staining in mouse lung tissues. Anti-CHI3L1 and -GATA3 antibodies were employed to stain lung tissues from *Rosa-CHI3L1^LSL/LSL^* and *Rosa-CHI3L1^LSL/LSL^* *IL-7R^cre^* mice. CHI3L1 is identified as red. GATA3 is identified as green and employed as a positive identifier of ILC2s. (**D**) Total bronchoalveolar lavage (BAL) collagen was quantified using a Sircol assay. (**E**) Representative histopathology of lung sections stained with H&E to depict the degree of fibrosis in *Rosa-CHI3L1^LSL/LSL^* and *Rosa-CHI3L1^LSL/LSL^* *IL-7R^cre^* mice that received bleomycin or vehicle (PBS). (**F** and **G**) Gene expression of COL-A1 mRNA and fibronectin mRNA was measured by RT-PCR. Values are mean ± SEM with 7–9 mice in each group. Comparisons between groups were conducted by 2-way ANOVA with Bonferroni’s post hoc test. *P ≤ 0.05; **P ≤ 0.01; ***P ≤ 0.001; ****P ≤ 0.0001. Scale bars: 100 μm.

To examine the cell-specific role of CHI3L1 expression, we generated *Rosa26* locus–targeted *CHI3L1*–conditional knockin transgenic mice (*Rosa-CHI3L1^LSL/LSL^*) that can be used to induce cell-specific overexpression when crossed with cell-specific promoter–driven Cre mice (Figure 9A). We bred *Rosa-CHI3L1^LSL/LSL^* mice with the *IL-7R^cre^* mice, in which CHI3L1 was specifically overexpressed only in the ILC2 population, and challenged them with bleomycin (Figure 9B) (52, 53). We confirmed CHI3L1 upregulation in GATA3^+^ cells, but not in alveolar macrophages, in *Rosa-CHI3L1^LSL/LSL^* mice when breeding with *IL-7R^cre^* mice (Figure 9C). *Rosa-CHI3L1^LSL/LSL^* and *Rosa-CHI3L1^LSL/LSL^* *IL-7R^cre^* mice were then subjected to i.p. bleomycin administration. Total BAL collagen was quantified using a Sircol assay. In these experiments, CHI3L1 overexpression in the ILC2 population increased the levels of bleomycin-induced collagen accumulation, α1 procollagen and fibronectin gene expression, and fibrosis development in WT and *Hps1*^−/−^ mice (Figure 9, D–G). Taken together, overexpression of CHI3L1 specifically in the ILC2 population using the *IL-7R^cre^* driver was sufficient to induce increased fibroproliferation.

## Discussion

Our previous studies demonstrate that CHI3L1 levels are elevated in HPS-1 and HPS-4 patients and that elevated levels of CHI3L1 are associated with disease progression. In addition, these studies demonstrate that exaggerated CHI3L1-driven fibroproliferation plays an important role in HPSPF, and that these divergent responses are mediated by differences in the trafficking and effector functions of CHI3L1 receptors. Specifically, the interaction between CHI3L1 and CRTH2, which occurs on various inflammatory cells, including ILC2s, drives the fibroproliferative responses (16, 17). However, the direct involvement of CHI3L1 and CRTH2 interaction on ILC2s and the crosstalk between ILC2s and fibroblasts that participate in HPSPF had not been defined. This work elucidates this complex interaction and thereby provides further insights into the mechanisms underlying pulmonary fibrosis in HPS, a prototypical disease model for fibrotic lung disease.

ILCs are a family of innate lymphocytes that are distinct from adaptive immune cells such as T and B cells (54–56). ILC2s play an important role in the pathogenesis of allergic asthma, as they coordinate with lung epithelium and interact with structural cells as well as other innate and adaptive immune cells (54, 55). Specifically, ILC2s contribute to the initiation and the maintenance of the type 2 immune responses by affecting eosinophilic lung inflammation, airway hyperresponsiveness, and mucus hypersecretion (39, 40). In addition, ILC2s stimulate M2 macrophage activation, skew dendritic cells toward a pro-Th2 phenotype, and enhance CD4^+^ T cell proliferation (57–59). Recent findings of increased pulmonary expression of IL-25 and numbers of ILC2s in the lungs of IPF patients prompt further research to clarify their roles in fibrotic lung disease (41). In IPF patients, Hams et al. observed increased expression of IL-25, and increased population of lineage marker–negative, CRTH2^+^T1/ST2^+^CD45^+^ICOS^+^IL-7Rα^+^IL-17BR^+^ ILC2 cells in the BAL. Murine studies have also shown that IL-13 release from ILC2s is sufficient to drive collagen deposition in the lungs of *Schistosoma* *mansoni* egg–challenged mice (41). In the bleomycin mouse model of lung fibrosis, ILC2s are activated by an IL-33/ST2–dependent mechanism and promote fibroblast activation (42, 43). Taken together, these studies have highlighted a unique role of ILC2 activation, and their interactions with fibroblasts in fibrotic lung diseases. However, it remains unclear through what mechanisms ILC2s accumulate in the lung under fibrotic conditions and whether lung ILC2 migration and activation contribute to HPSPF.

Previous studies have demonstrated that lineage-negative, CRTH2^+^T1/ST2^+^CD45^+^ICOS^+^IL-7Rα^+^IL-17BR^+^ ILC2s were increased in the BAL of IPF patients compared with control patients (41–43). We expanded these findings by demonstrating that ILC2s can be identified in the lungs of IPF and HPS patients. As fresh lung tissues for this extremely rare lung disease that could be used for flow cytometry analyses are not accessible, technically we are limited by the availability of reliable markers and antibodies to identify ILC2s. Since ILC2s have multiple cell surface markers that overlap with other immune cell populations, we decided to label them as GATA3^+^CD90^+^CD3^–^CD20^–^CD79α^–^CD56^–^ cells. As noted in Figure 1 and Supplemental Figure 1, the numbers of ILC2s were significantly increased in HPSPF patients compared with IPF patients and normal individuals, and the GATA3^+^Lineage^–^ cells (including ILC2s) appeared to be in close proximity to the fibrotic foci. ILC2s are known to skew dendritic cells toward a pro-Th2 phenotype and enhance CD4^+^ T cell proliferation (57–59). It is possible that the interactions between ILC2s and innate and adaptive type 2 immune responses are involved in activating the Th2s in both IPF and HPSPF patients. It must be noted that we only examined a small cohort of HPSPF lung tissues for the current study because HPS is a rare disorder and only limited tissue specimens are available. Further studies with a larger cohort of both IPF and HPSPF patients will be needed to confirm these findings.

Our previous results demonstrated that, in *Hps1*^−/−^ mice, exaggerated collagen accumulation in the lung is mediated by CHI3L1 interaction with CRTH2, and CRTH2 inhibition significantly diminishes this CHI3L1-induced fibrotic response (16, 17). CRTH2, a G protein–coupled receptor known for binding with prostaglandin D2 (PGD2), plays an important role in the pathogenesis of allergic Th2 inflammation in the lung (60, 61) and fibrosis development in the kidney (62–64). The expression of CRTH2 is commonly used to identify ILC2s in the lung (31–33). Our follow-up studies demonstrated that CHI3L1 and CRTH2 physically bind one another, and CRTH2 inhibition significantly diminishes ILC2 migration in the lungs of *Hps1*^−/−^ mice. Two methods of ILC2 depletion were employed to determine the contribution of ILC2s in HPS-lung fibrosis: an IL-33 siRNA to knock down the expression of IL-33 and *Rora^fl/fl^* *IL-7R^cre^* mice, in which ILC2s are depleted (49–51). In both models, depletion of ILC2s significantly diminished fibrosis development in the *Hps1*^−/−^ mice, suggesting that ILC2-mediated mechanisms contributed to optimal CHI3L1-induced amplified fibrotic responses in the bleomycin-induced pulmonary fibrosis in *Hps1*^−/−^ mice.

ILC2s are able to rapidly produce large amounts of type 2 cytokines and growth factors, such as IL-5, IL-13, and AREG (34–36) to initiate type 2 immune responses, induce eosinophilic lung inflammation, airway hyperresponsiveness, mucus hypersecretion (39, 40), and to drive healing and fibrotic repair (35). In order to determine what cytokines and growth factors released from ILC2s contribute to the development of lung fibrosis in HPS, we sorted Lineage^–^ (CD3^–^CD11b^–^CD45R/B220^–^Ly76^–^Ly6G^–^Ly6C^–^), Thy1.2^+^ICOS^+^T1/ST2^+^ ILC2s and examined the gene expression by real-time PCR and RNA-Seq. We found that IL-5, IL-13, and AREG were significantly increased in sorted ILC2s from bleomycin-challenged *Hps1*^−/−^ mice, and indeed the AREG/EGFR pathway was partially responsible for increased fibroblast proliferation and differentiation in the coculture experiments. In addition, many genes responsible for matrix remodeling and collagen production were also increased and Reactome analysis highlighted that immune system and extracellular matrix organization were the 2 hubs of genes altered in ILC2s isolated from *Hps1^−/−^* mice compared with WT mice upon bleomycin treatment (Figure 7E). Interestingly, even though levels of IL-5 and IL-13 were increased in the media of ILC2 culture from *Hps1*^−/−^ mice, levels of eosinophilia and eotaxin expression were comparable, and no asthma-related phenotypes were observed in these mice after bleomycin challenges. It is possible that bleomycin is not able to induce typical type 2 immune response–mediated lung fibrosis, or that ILC2s contribute to fibrosis development through other pathways, including the EGFR pathway that was investigated above. This is further supported by the fact that when challenged with HDM extract to induce typical type 2 immune responses, *Hps1*^−/−^ mice had increased numbers of eosinophils as well as eotaxin mRNA levels in the lung.

Among the genes that were altered in the ILC2s from *Hps1*^−/−^ mice, we also examined the expression of CHI3L1 and its receptor CRTH2. Interestingly, our data demonstrated that ILC2s from *Hps1*^−/−^ mice express higher levels of CHI3L1 and CRTH2, suggesting they are primed to be activated by the CHI3L1/CRTH2 axis. When we used *IL-7R^cre^* to specifically overexpress CHI3L1 in ILC2 populations, we demonstrated that overexpression of CHI3L1 specifically in the ILC2 population using the *IL-7R^cre^* driver was sufficient to induce increased fibroproliferation. We acknowledge that the *IL-7R^cre^* mice studied may not be completely ILC2 specific, as T cells in adaptive immunity, specifically Th2s, are likely the other cell type that may be involved when using *IL-7R^cre^*. Nevertheless, our results indicate that CRTH2 expression on ILC2s is responsible for CHI3L1-induced recruitment and migration of ILC2s in the lung, which interact with fibroblasts and contribute to development of lung fibrosis.

Overall, our studies strongly demonstrated that CHI3L1 and its receptors play an important role in mediating pulmonary injury and repair in HPS, and targeting these moieties may provide novel and effective therapeutic options. Further experiments are needed to test this innovative hypothesis. Importantly, our studies of HPS provide insights into CHI3L1 biology and the mechanisms of protein trafficking that may underlie other fibrotic diseases like IPF.

## Methods

### Sex as a biological variable.

Our study examined male and female animals, and similar findings are reported for both sexes.

### Human tissue samples characteristics and ethical statements.

To identify type 2 ILCs and to elucidate their potential role in the pathobiology of pulmonary fibrosis, human lung tissues in normal individuals, IPF patients (Lung Tissue Research Consortium), and HPSPF patients were used in these evaluations. Patients with HPSPF provided written informed consent and were enrolled in protocol 04-HG-0211 (ClinicalTrials.gov NCT00084305, Analysis of Specimens from Individuals with Pulmonary Fibrosis). The HPS-1 human lung samples were obtained from molecularly confirmed adult HPS-1 patients with known pulmonary fibrosis based on high-resolution CT evaluation. Lung explants from HPS-1 patients with pulmonary fibrosis were collected when patients underwent clinically indicated lung transplantation, and tissue samples were fixed in formalin and sectioned. Study procedures and a Material Transfer Agreement (MTA) covering the transferred encoded specimens were approved by Brown University and the NHGRI. The IPF and normal control samples were obtained from the Lung Tissue Research Consortium.

### Immunofluorescent staining.

Immunofluorescent staining was conducted on 15 paraffin-embedded lung samples (5 controls, 5 IPF, and 5 HPSPF) after dewaxing in xylene and rehydration with graded alcohol series. ILC2s are characterized by the expression of GATA3 (65). To identify and localize ILC2s, GATA3 staining was undertaken on sections along with antibodies against human NK cells, B cells/plasma cells, T cells, and α-SMA. To avoid bias, ensure accuracy, and minimize potential errors in quantification, we carefully selected multiple images (normalized per area) from the tissue segments and screened them, rejecting images based on the presence of certain biological structures (e.g., too many blood vessels, lymphatic vessels, and airways), as well as if there is an area out of the section in the image (e.g., empty space or in the pleural space). The following primary antibodies (dilution; catalog/clone number, manufacturer) were used for overnight incubation at 4°C: GATA3 (1:250; ab199428, Abcam), CD90 (1:200; AF2067, R&D Systems), CD56 (1:200; ab9272, Abcam), CD20 (1:100; IGEL/773, Novus), CD79A (1:100; NB100-64347ss, Novus), CD-3e (4:100; ab699, Abcam), and α-SMA (1:200; NB300–978, Novus). The slides were then washed and incubated with secondary antibodies Alexa Fluor 594 (1:200; ab150080; Abcam), Alexa Fluor 488 (1:200; ab150077, Abcam), and Alexa Fluor 405 (1:200; A48259, Invitrogen) for 60 minutes at room temperature. PBS was used as a washing buffer between incubation steps. Finally, the sections were stained and mounted with Vectashield containing 4′,6-diamidino-2-phenylindole (DAPI) (50 μL; H-1200, VECTOR Laboratories) or mounted with Cytoseal XYL mounting media (Richard Allan Scientific). Fluorescence was detected by immunofluorescence microscopy using a Leica DMI6000 inverted epifluorescence microscope.

### WT, knockout, conditional knockout, and conditional knockin transgenic mice.

WT (C57BL/6) and *HPS1*^−/−^ mice were obtained from The Jackson Laboratory. *HPS1*^−/−^ mice were bred with *CRTH2*^−/−^ mice to generate *HPS1*^−/−^ *CRTH2*^−/−^ double-mutant mice. ILC2-depleted *Rora^fl/fl^* *IL-7R^cre^* mice were a gift from Andrew Mckenzie at the MRC Laboratory of Molecular Biology (Cambridge, United Kingdom) and Hans-Reimer Rodewald at the German Cancer Research Center (Heidelberg, Germany). *Rora^fl/fl^* *IL-7R^cre^* mice were then bred with *HPS1*^−/−^ mice to generate *HPS1*^−/−^ *Rora^fl/fl^* *IL-7R^cre^* mice. *Rosa26* locus–targeted *CHI3L1*–conditional knockin transgenic mice (*Rosa-CHI3L1^LSL/LSL^*) were generated at the Brown Mouse Transgenic and Gene Targeting Facility. *Rosa-CHI3L1^LSL/LSL^* mice were also bred with *IL-7R^cre^* mice to generate ILC2-specific *CHI3L1*-overexpressing mice (*Rosa-CHI3L1^LSL/LSL^* *IL-7R^cre^*). All mice were genetically modified congenic on a C57BL/6 background and were genotyped as previously described (49–51).

### Intratracheal bleomycin treatment.

WT, *HPS1*^−/−^, and *HPS1*^−/−^ *CRTH2*^−/−^ double-mutant mice were exposed to a single bleomycin injection (1.25 U/kg; Teva Parenteral Medicines) via i.t. administration diluted in 50 μL sterile PBS or PBS alone. Mice were anesthetized with isoflurane prior to i.t. administration. Mice were sacrificed on day 14 after bleomycin instillation. Clodronate depletion of intrapulmonary macrophages in the lung were achieved by treating mice with liposomal clodronate 5 days after bleomycin administration every 3 days. For IL-13 blockade, mice were treated with IL-13–blocking antibody (100 μg/treatment, every other day from day 6 to 14).

### Intraperitoneal bleomycin treatment.

Ten-week-old WT, *HPS1*^−/−^, *Rora^fl/fl^* *IL-7R^cre^*, *HPS1*^−/−^ *Rora^fl/fl^* *IL-7R^cre^*, *Rosa-CHI3L1^LSL/LSL^*, and *Rosa-CHI3L1^LSL/LSL^* *IL-7R^cre^* mice were treated with either PBS or bleomycin (25 U/kg) in PBS by i.p. injection in a total volume of 100 μL. This treatment was repeated daily for 6 consecutive days. Mice were sacrificed on day 12.

### HDM model.

In the HDM model, mice were anesthetized with isoflurane prior to intranasal (i.n.) administration of either 25 μL PBS (control) or 25 μL (1 mg/ml in PBS) of purified HDM, *Dermatophagoides* *pteronyssinus* (Der P1), extract (Greer Laboratories) 3 times a week for 3 weeks and sacrificed 18–24 hours after the last HDM exposure.

### Preparation of single-cell suspensions.

Harvested lungs were rinsed with cold PBS and minced with scalpels prior to digestion. Lung tissue was placed in a gentleMACS C Tube (Miltenyi Biotec, 130-093-237) and digested using the lung dissociation kit enzymes (Miltenyi Biotec, 130-095-927) and the gentleMACS Dissociator (Miltenyi Biotec, 130-093-235), as per the manufacturer’s recommendation. Cells were passed through a 70-μm cell strainer and treated with cold ACK lysis buffer (155 mM NH_4_Cl, 9.99 mM KHCO_3_, 0.10 mM EDTA) to remove RBCs. The cells were then washed and stained with various antibodies conjugated with fluorochromes (see next section).

### Flow cytometry.

APC-conjugated anti-Lineage markers, PE-conjugated anti-ICOS, FITC-conjugated anti-CD90.2, and PE-Cy7–conjugated anti-T1/ST2 were obtained from BD Pharmingen (catalog 558074) and eBioscience (catalog 12-9949-81, 11-0903-82, and 25-9335-82). Flow cytometry was performed using a BD FACSAria. ILC2s are defined as Lineage^–^ (CD3^–^CD11b^–^CD45R/B220^–^Ly76^–^Ly6G^–^Ly6C), Thy1.2^+^ICOS^+^T1/ST2^+^ cells. Data were analyzed using FlowJo software v.10 (TreeStar Inc.). Percentages of Lineage^–^Thy1.2^+^ICOS^+^T1/ST2^+^ cells and total cells recovered from the lungs were used to determine the extent of ILC2 recovery. For all analyses, isotype control staining was subtracted from specific antibody staining to determine the percentage of positive cells.

### ILC2 culture.

ILCs were enriched from single-cell suspensions of mouse lungs by negative selection using Stemcell EasySep Mouse Pan-ILC Enrichment Kit. Lineage^–^Thy1.2^+^ICOS^+^T1/ST2^+^ ILC2s were sorted on a BD FACSAria to a purity of 98% or greater. Lung ILC2s sorted from WT and *HPS1^−/−^* mice were plated on 6-well plates. Sorted ILC2s were cultured in RPMI 1640 supplemented with 10% FBS and IL-2 (10 ng/mL; R&D Systems, 1150ML020). Total RNA was then extracted and used for RNA-Seq and gene expression analyses. The number of ILC2s used in sequencing were WT PBS, 22,405 cells; HPS PBS, 38,873 cells; WT bleomycin, 84,166 cells; and HPS bleomycin, 128,647 cells.

### ILC2 migration assay.

Primary ILC2s from the lungs of WT mice were enriched and sorted as described above. Sorted lung ILC2s were allowed to rest for 2 hours at 37°C in media. Media with 500 ng/mL CHI3L1 was placed in the bottom chamber of Costar 6.5 mm, 5.0 μm Transwell 24-well plates, with or without the presence of CRTH2 inhibitor CAY10471 (Cayman Chemical), and 5 × 10^4^ ILCs were placed in the top chamber. The numbers of migrating cells were quantified using CountBright absolute counting beads (Thermo Fisher Scientific, CC36950) by flow cytometry analysis.

### CRTH2 inhibitor treatment in vivo.

WT and *HPS1*^−/−^ mice were treated with CRTH2 inhibitor (CAY10471). Each injection contained 0.4 mg of the CAY10471 compound dissolved in 15.5 μL ethanol, 7.7 μL DMSO, 15.5 μL polyethylene glycol 400, and 33.3 μL PBS, resulting in a volume of 72 μL per injection. Mice were administered i.p. injections twice per week of either the drug formulation or PBS.

### IL-33 siRNA treatment.

WT and *HPS1*^−/−^ mice were treated with IL-33 siRNA (every other day, 3 nmol/mouse) or its scrambled control to allow fibrotic tissue repair responses, and lung ILC2s were harvested as described above.

### Quantification of lung collagen.

Animals were anesthetized using 75–1000 mg/kg urethane i.p. Median sternotomy was performed and right heart perfusion was completed with calcium- and magnesium-free PBS. The heart and lungs were then removed. The right lung was frozen in liquid nitrogen and stored at –80°C until used. Collagen content was determined by quantifying total soluble collagen using the Sircol Collagen Assay kit (Biocolor, Accurate Chemical & Scientific Corp.) according to the manufacturer’s instructions.

### Gene expression analysis.

Cells processed from mouse lungs were lysed in TRIzol reagent (Invitrogen) and total cellular RNA was extracted by Qiagen RNeasy kit per manufacturer’s instructions. From the mRNA, cDNA was synthesized using the Bio-Rad iScript cDNA Synthesis kit per manufacturer’s instructions. The corresponding mRNA level was then measured using real-time PCR. The primer sequences for IL-5, IL-13, AREG, CRTH2, CHI3L1, COL-A1, fibronectin 1 (Fn-1), and GAPDH were obtained from PrimerBank (http://pga.mgh.harvard.edu/primerbank/). The sequences are IL-5-F, TCAGGGGCTAGACATACTGAAG; IL-5-R, CCAAGGAACTCTTGCAGGTAAT; IL-13-F, ATGCCCAACAAAGCAGAGAC; IL-13-R, TGAGAGAACCAGGGAGCTGT; CRTH2-F, CCTTTTTTCCACCTTGCCATG; CRTH2-R, CCAGGATAGTTGGCATGTC; CHI3L1-F, GTACAAGCTGGTCTGCTACTTC; CHI3L1-R, ATGTGCTAAGCATGTTGTCGC; AREG-F, CATTATGCAGCTGCTTTGGA; AREG-R, GTCGTAGTCCCCTGTGGAGA; COL-A1-F, GCTCCTCTTAGGGGCCACT; COL-A1-R, CCACGTCTCACCATTGGGG; Fn1-F, ATGTGGACCCCTCCTGATAGT; Fn1-R, GCCCAGTGATTTCAGCAAAGG; GAPDH-F, AGGTCGGTGTGAACGGATTTG; and GAPDH-R, TGTAGACCATGTAGTTGAGGTCA.

### ELISA.

Levels of soluble mediators CHI3L1 and AREG were assayed using commercially available ELISA kits (R&D) following the manufacturer’s instructions.

### Isolation and culture of primary lung fibroblasts.

Primary mouse lung fibroblasts were isolated from the lung tissues of WT and *HPS1^−/−^* mice. In brief, mice were anesthetized as described above. The lung tissues were collected and minced using a scalpel and digested with 0.14 Wunsch units/mL Liberase Blendzyme 3 (Sigma-Aldrich, 5401119001), and 1× antibiotic/antimycotic at 37°C for 60 minutes. After centrifuging at 524*g* for 5 minutes, the pellet was resuspended with DMEM/F12 media with 15% FBS, 1× antibiotic/antimycotic, and cultured into each well of a 6-well plate and at 37°C, 5% CO_2_, and 3% O_2_ for 1 week. Then, fresh media were added, and the cells were passaged and incubated for an additional 7 days, until cells reached 80%–90% confluence. Then, the cells were passaged and cultured in a new 4-well chamber slide and 96-well plates, 48–24 hours before ILC2 sorting.

### ILC2 and primary lung fibroblast coculture.

Sorted primary ILC2s from bleomycin- and PBS-treated WT and *HPS1^−/−^* mice were added to the primary fibroblast culture in 4-well chamber slides and 96-well plates followed by 10 μM gefitinib (Tocris Bioscience) to block AREG signaling. Cocultured ILC2s and fibroblasts were incubated under standard conditions in 4-well chamber slides and 96-well plates for 4 days.

### BrdU and α-SMA immunofluorescent staining.

BrdU solution in PBS (33:1000; BD Pharmingen, 550891) was added to the ILC2 and fibroblast coculture. Cell cultures were then incubated for 24 hours at 37°C, followed by fixation in 4% paraformaldehyde for 15 minutes. Cells were then permeabilized in 0.2% Triton X-100 (Sigma-Aldrich) for 15 minutes, and incubated with 2% BSA for 1 hour. For BrdU staining, cells were treated with mouse anti-BrdU antibody (555627, BD Pharmingen) overnight at 4°C. For α-SMA staining, slides were incubated with an α-SMA mAb (1:100; clone 1A4, Sigma-Aldrich) overnight at 4°C. After a wash step with PBS, cells were incubated with secondary antibodies for 60 minutes at room temperature. For BrdU, Alexa Fluor 488 (1:200; ab150077, Abcam), and for α-SMA, Alexa Fluor 405 (1:200; A48259, Invitrogen) were used. Finally, the slides were stained and mounted with DAPI. Fluorescence-positive cells in each group were observed under a fluorescence microscope as described above.

### BrdU assay for DNA replication and cell division.

BrdU, a synthetic thymidine analog, can be incorporated into newly synthesized DNA, providing a test of DNA replication, as an indirect measure of cell division. The assay was performed as described in the product (catalog 2750) manual from Millipore. BrdU incorporation was detected by addition of peroxidase substrate. Spectrophotometric detection was performed at a wavelength of 450 nm.

### BAL and histology.

Mice were anesthetized and airways were lavaged 3 times with 0.3 mL PBS, and 1 mL BAL fluid was recovered and stored at –80°C until used. After lavage, the lungs were inflated with 10% buffered formalin at 25 cm of pressure and fixed at 4°C overnight. Lungs were dehydrated in ethanol gradients and embedded in paraffin, and 5-μm tissue sections were collected on microscope slides and stained with H&E according to manufacturer’s instructions (Epredia). Histopathologic assessment of lungs was conducted, and BAL collagen content was determined by quantifying total soluble collagen using the Sircol Collagen Assay kit as described above.

### Animal study approval.

Animal experiments were approved by the IACUC of Brown University in accordance with federal guidelines.

### Statistics.

Mouse data are expressed as mean ± SEM. As appropriate, groups were compared by ANOVA with Bonferroni’s post hoc test; follow-up comparisons between groups were conducted using a 2-tailed Student’s *t* test. A *P* value of 0.05 or less was considered to be significant. Statistical analysis was performed using Prism (GraphPad Software Inc.). Graphs were generated using Microsoft Excel and Prism.

### Data availability.

All raw data, including those for graphs and reported means, are provided in the supplemental Supporting Data Values Excel file. The next-generation sequencing data generated for this study have been deposited in the NCBI GEO under accession number GSE278788.

## Author contributions

PS, KKS, SS, DY, and YZ designed the research studies. PS, KK, SS, DY, and YZ conducted experiments. PS, KKS, SS, DY, and YZ acquired data. PS, KKS, SS, DY, and YZ analyzed data. AXY, CN, WJI, and BRG provided reagents. PS, KKS, BRG, and YZ wrote the manuscript.

## Supplementary Material

Supplemental data

Supporting data values

## Version 1 10/15/2024

In-Press Preview

## Version 2 11/22/2024

Electronic publication
