# Supplementary material for: Type 2 innate immunity promotes the development of pulmonary fibrosis in Hermansky-Pudlak syndrome
Source: JCI Insight. 2024 Nov 22;9(22):e178381. doi: 10.1172/jci.insight.178381 (PMC11601950; doi:10.1172/jci.insight.178381)
Supplement: Supplemental data [file jciinsight-9-178381-s159.pdf]

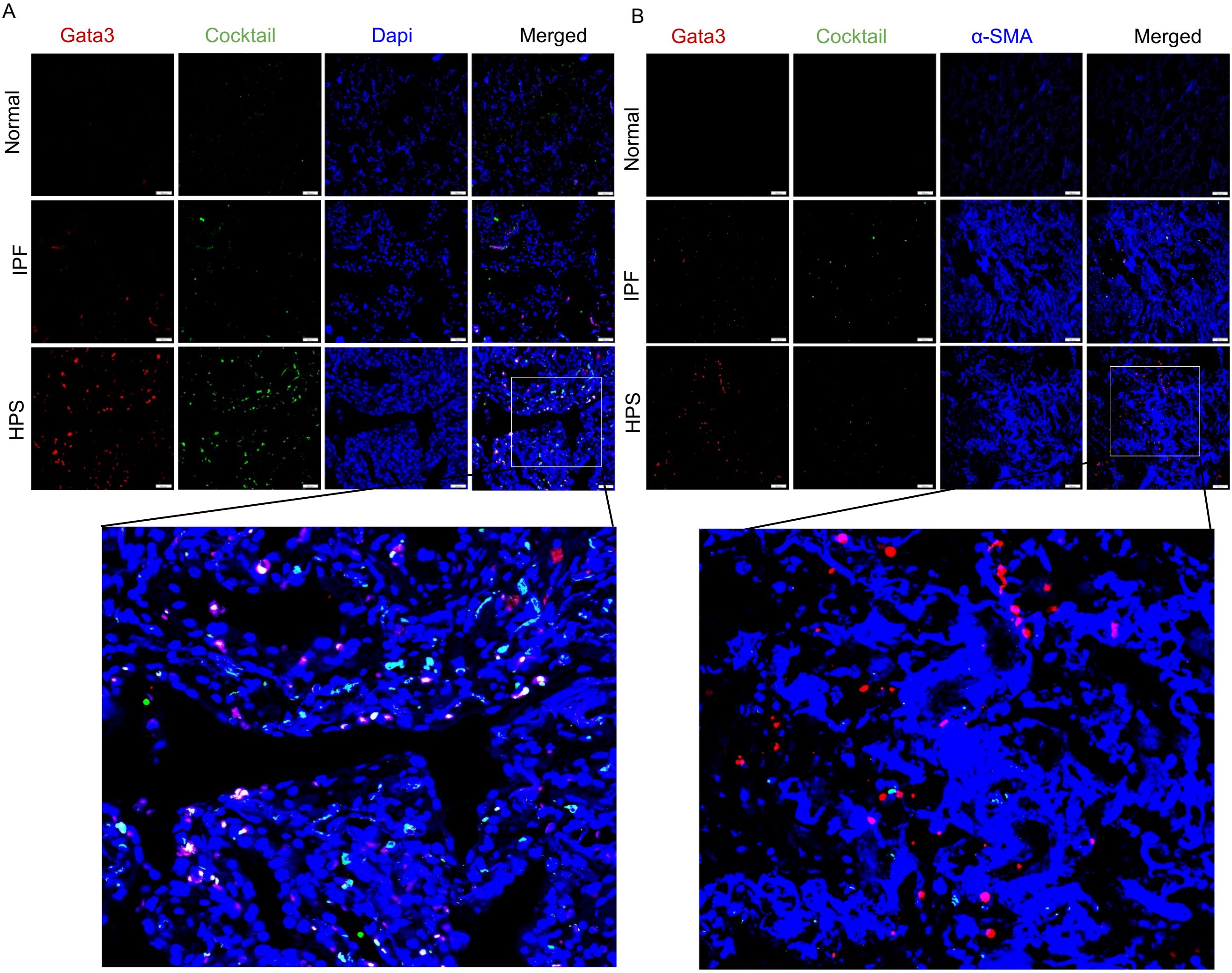


Supplemental Figure 1. ILC2s are increased in human HPS and they are found in the fibrotic region. (A) ILC2 staining in human lung tissues. Gata3 and a cocktail of 3 antibodies including CD3, CD56 and CD20/79 were employed to stain human lung tissues in normal individuals, idiopathic pulmonary fibrosis (IPF) patients, and HPS patients. ILC2s are identified as Gata3 positive and antibodies cocktail negative cells. (B) ILC2 and α-SMA staining in human lung tissues. Gata3, cocktail of 3 antibodies including CD3, CD56 and CD20/79 and a-SMA were employed to stain human lung tissues in normal individuals, idiopathic pulmonary fibrosis (IPF) patients, and HPS patients. ILC2s are identified as Gata3 positive and antibodies cocktail negative cells. α-SMA is identified as cascade blue staining.

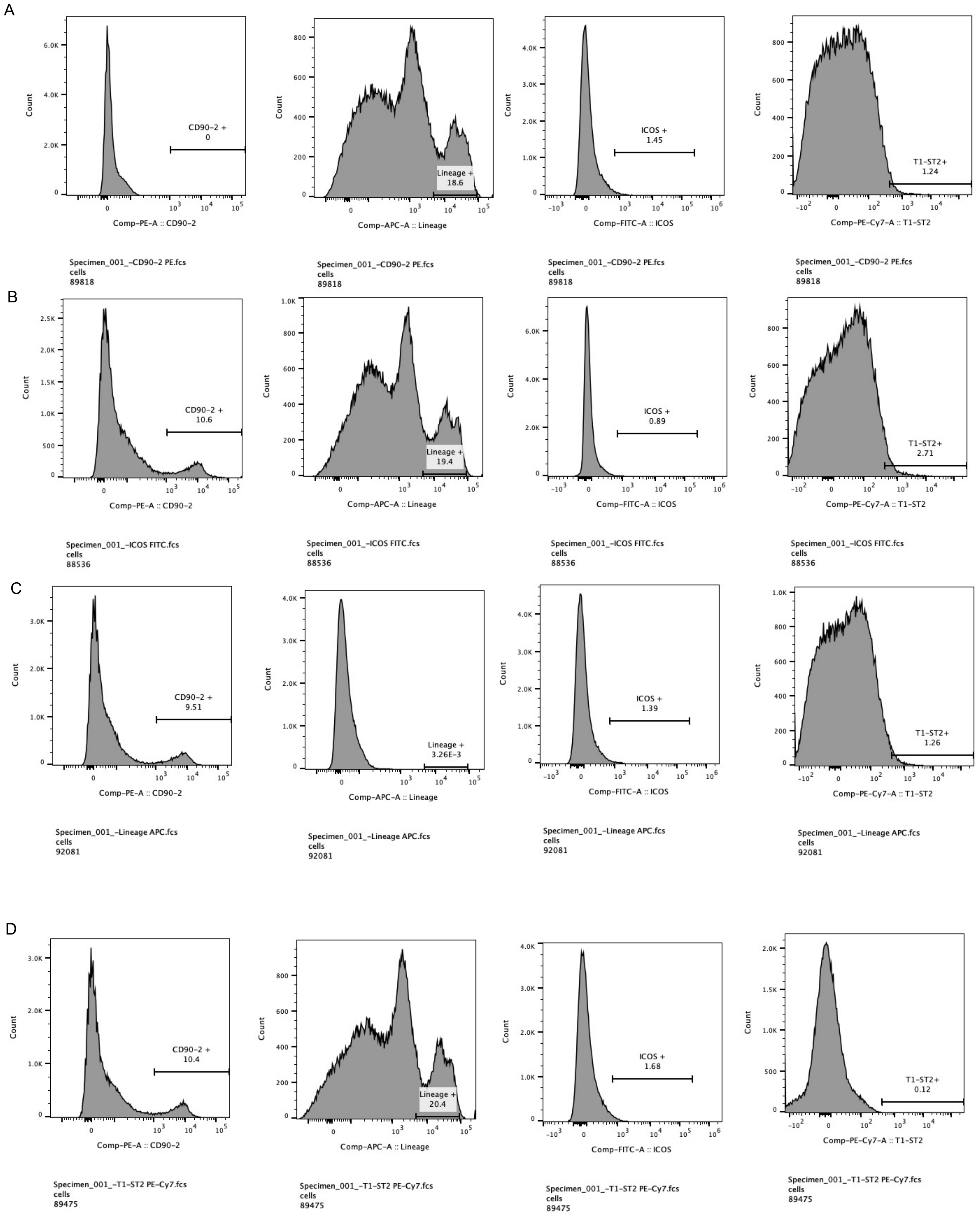


Supplemental Figure 2. Fluorescence minus one (FMO) control tubes for mouse ILC2 gating. (A) Cells were stained with Lineage makers, ICOS, and T1-ST2, but not CD90.2. (B) Cells were stained with Lineage makers, CD90.2, and T1-ST2, but not ICOS. (C) Cells were stained with ICOS, and T1-ST2, CD90.2, but not Lineage makers. (D) Cells were stained with Lineage makers, ICOS, and CD90.2, but not T1-ST2.

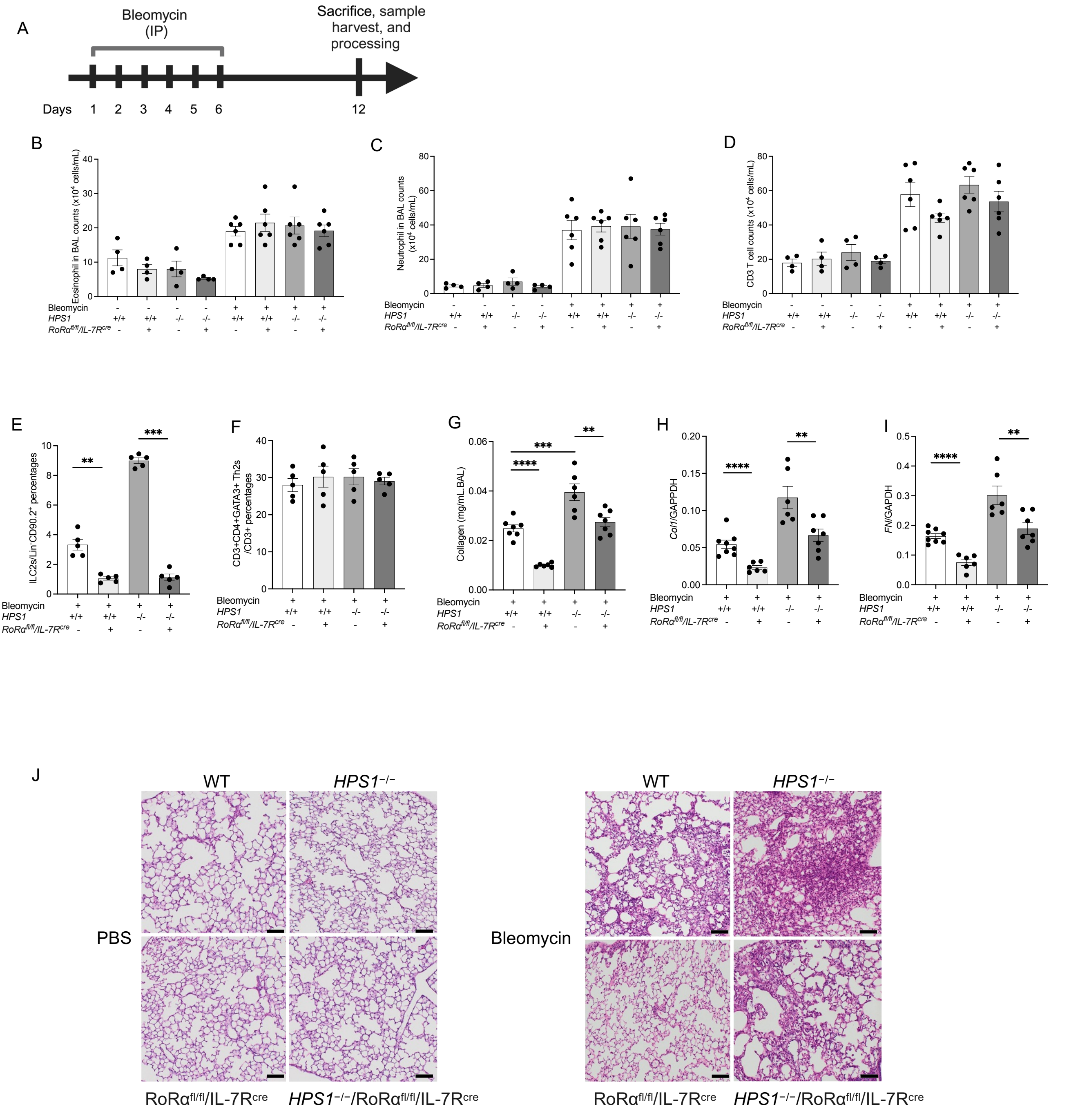


<u>Supplemental Figure 3.</u> ILC2 depletion diminishes HPS/bleomycin-induced lung fibrosis. RoRα$^{fl/fl}$/IL-7R$^{cre}$ mice, in which ILC2s are depleted, were bred with *HPS1*$^{-/-}$ mice, and mice were subjected to bleomycin administration. WT and HPS1-/- mice were subjected to intratracheal bleomycin administration. (A) Schematic of the experiment. (B) to (D) Numbers of BAL eosinophils, neutrophils, and T cells were not altered by ILC2 depletion. (E) and (F) Percentages of lung ILC2s and Th2s were assessed by flow cytometry. (G) Total lung collagen was quantified using Sircol assay on Day 14. (H) and (I) Gene expression of COL-A1 mRNA and fibronectin mRNA was measured by RT-PCR. (J) Representative histopathology lung sections stained with H&E to depict the degree of fibrosis. Values are mean± SEM with 7-9 mice in each group. Comparisons between groups were conducted by two-way ANOVA with Bonferroni's post test. *p ≤ 0.05, **p ≤ 0.01, ***p ≤ 0.001, and ****p ≤ 0.0001. Scale bar, 100µm.

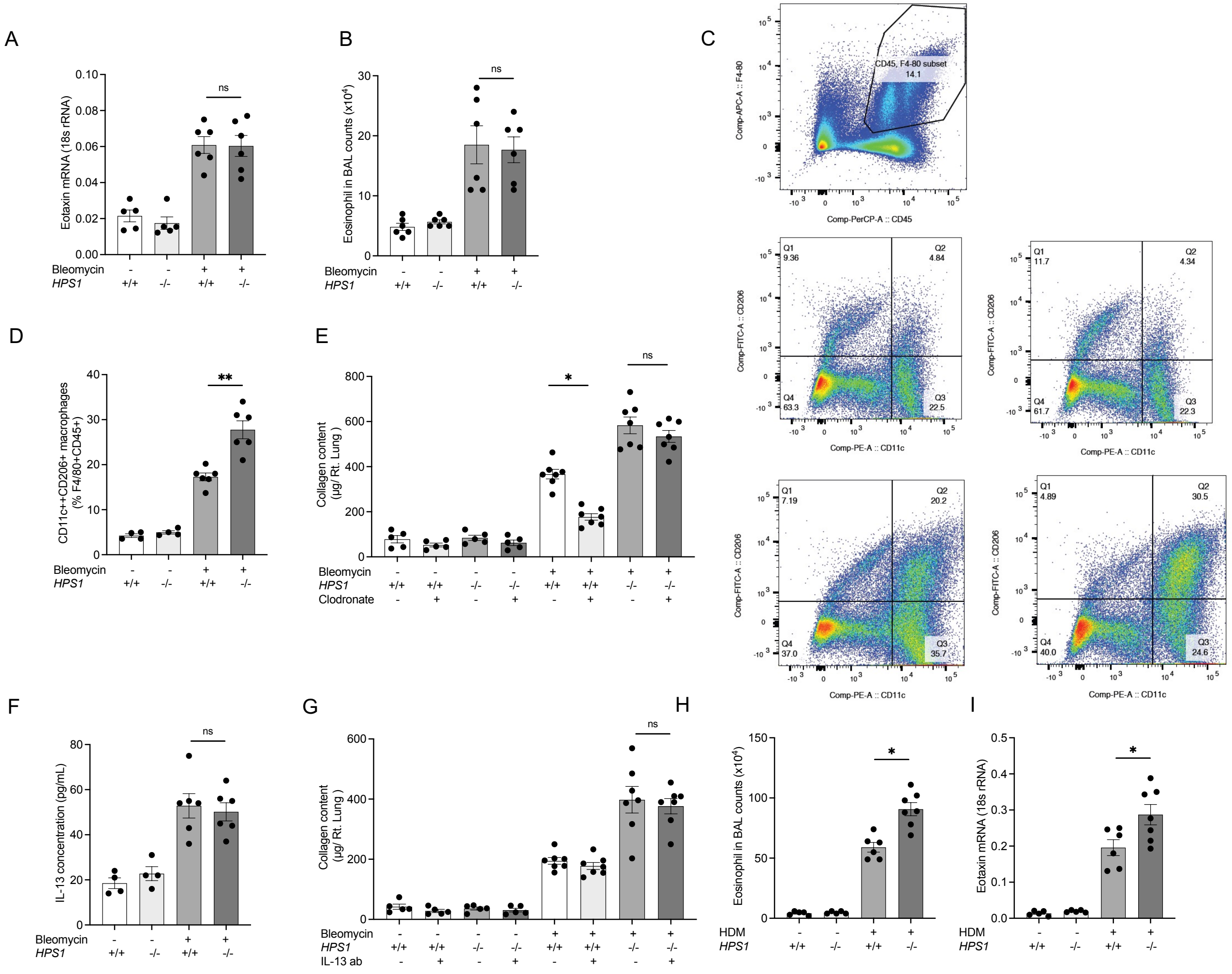


Supplemental Figure 4. Characterizing eosinophils, macrophages, and the role of IL-13 in HPS lung fibrosis. WT and HPS1 mice were subjected to intratracheal bleomycin administration. (A) BAL eosinophils were counted by Diff-Quik staining. Lungs were digested, and (B) mRNA levels of eotaxin were quantified. (C) and (D) FACS analysis was used to analyze CD11c and CD206 expression on CD45+F4/80+ gated cell populations. (E) Mice were treated with clodronate liposomes to deplete macrophages, and Sircol assay was used to measure the amount of collagen accumulation in the lung. (F) BAL IL-13 levels were quantified by ELISA. (G) Mice were treated with IL-13 blocking antibody (100ug/treatment, every other day from day 6 to 14), and Sircol assay was used to measure the amount of collagen accumulation in the lung. (H) to (I) WT and HPS1 mice were subjected to HDM challenges. BAL eosinophils were counted by Diff-Quik staining (H). Lungs were digested, and mRNA levels of eotaxin were quantified (I). The noted values are the mean ± SEM of evaluations of 4-7 mice in each group. Comparisons between groups were conducted by two-way ANOVA with Bonferroni's post test. $^{**}p \le 0.01$. $^{*}p \le 0.05$.

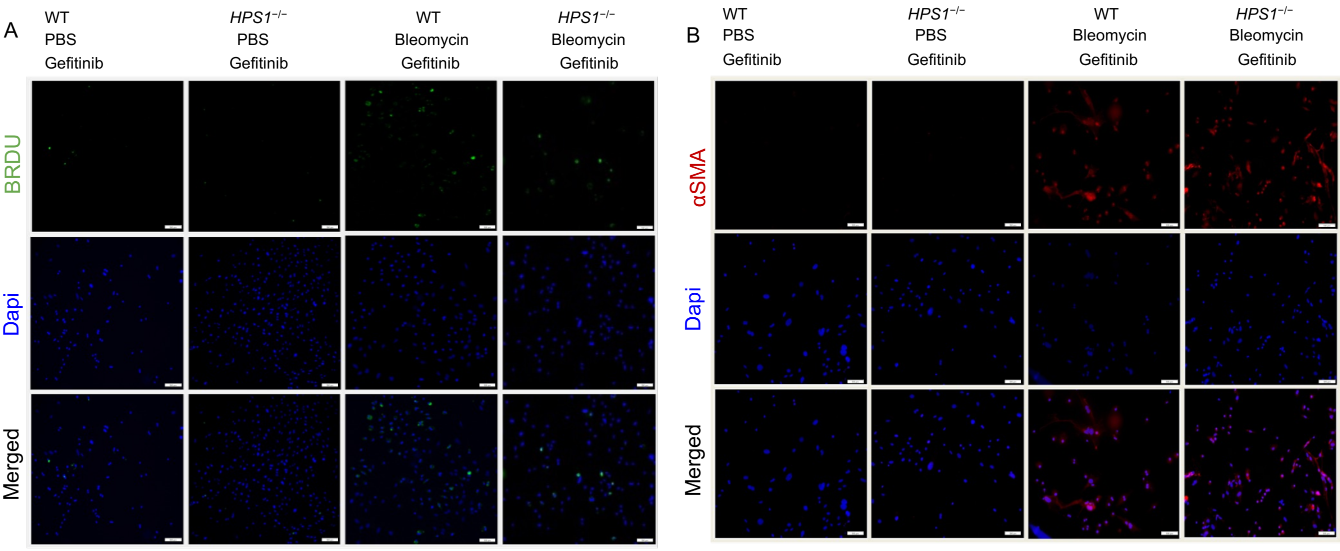


<u>Supplemental Figure 5.</u> WT and *HPS1-/-* mice were subjected to IP bleomycin administration. primary ILC2s were sorted from mouse lung and co-cultured with primary fibroblast. Gefitinib was added to the cultured media. Blocking Areg signaling utilizing gefitinib significantly reduced the ability of ILC2s to stimulate fibroblast proliferation (A) and expression of α-SMA (B). Images were representative of 4 independent experiments. Scale bar, 100μm.

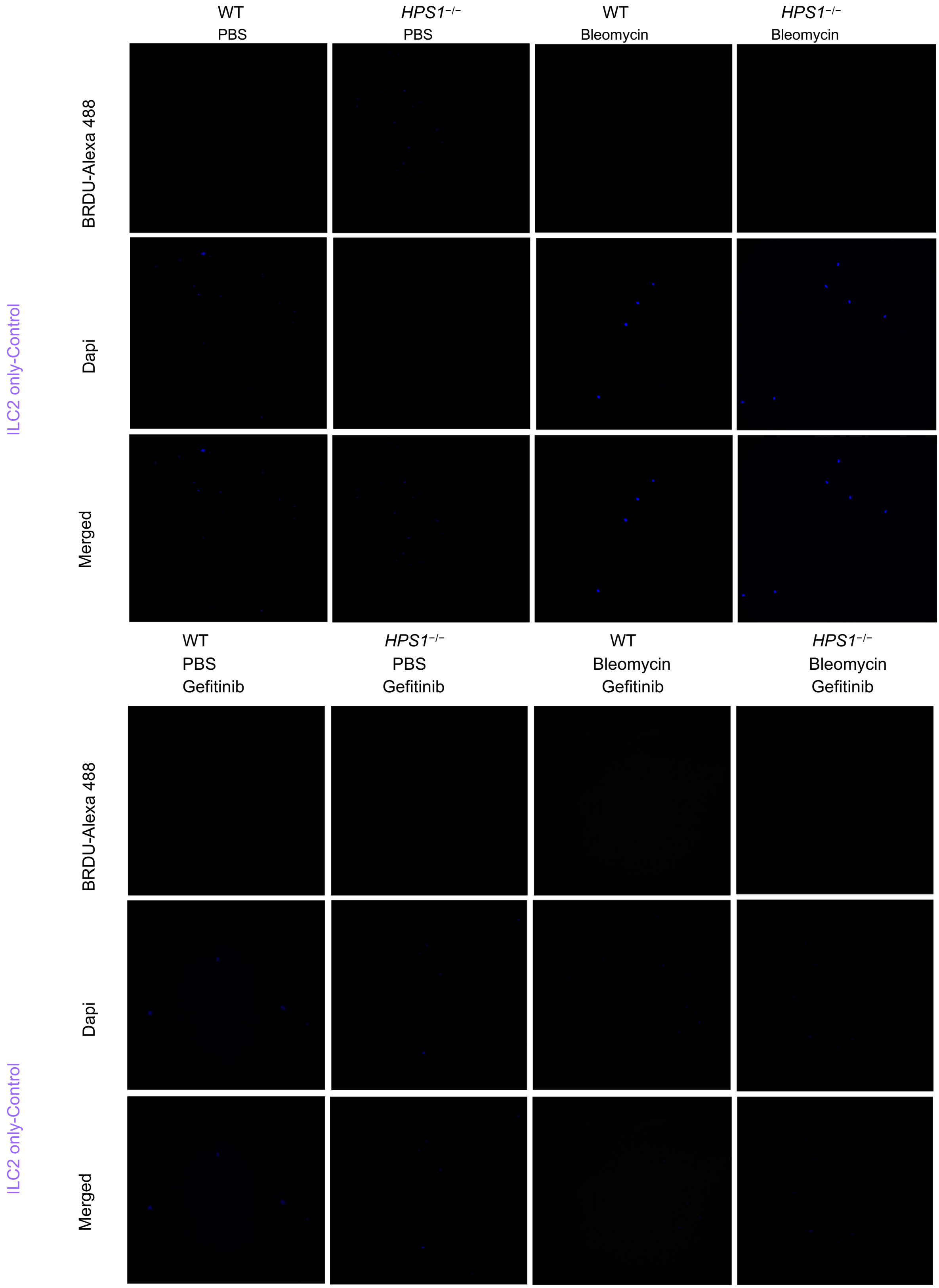


Supplemental Figure 6. WT and *HPS1-/-* mice were subjected to IP bleomycin administration. Primary ILC2s were sorted. ILC2s only from PBS and bleomycin treated WT and *HPS1-/-* mice. BrdU immunostaining was used to identify proliferating cells. (magnification, x40). Images were representative of 4 independent experiments.

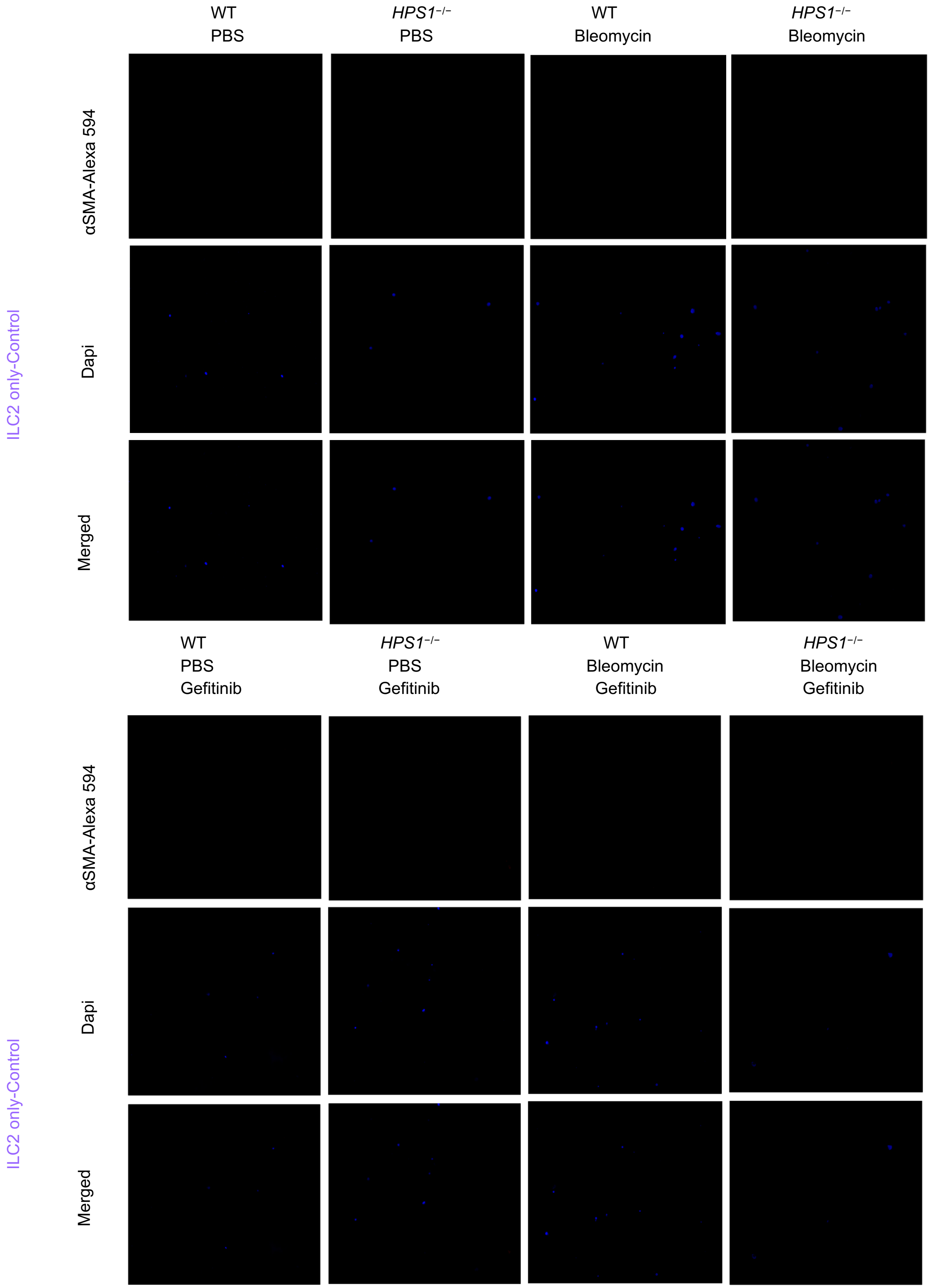


Supplemental Figure 7. WT and *HPS1-/-* mice were subjected to IP bleomycin administration. Primary ILC2s were sorted from mouse lung. ILC2s only from PBS and bleomycin treated WT and *HPS1-/-* mice. α-smooth muscle actin (α-SMA) Immunostaining was was used to detect α-SMA expression (magnification, x40). Images were representative of 4 independent experiments.

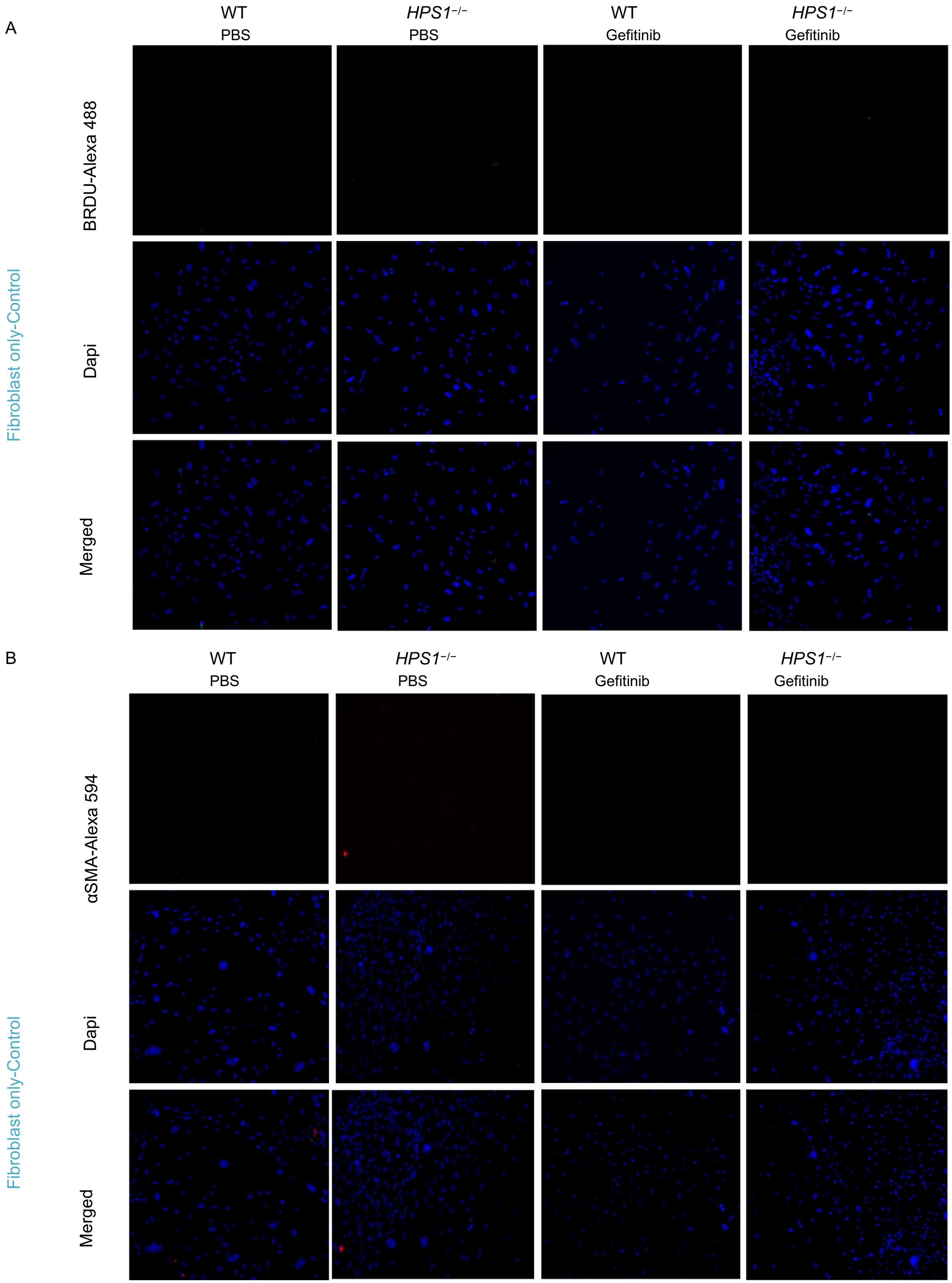


Supplemental Figure 8. WT and *HPS1-/-* mice were subjected to IP bleomycin administration. Primary ILC2s were sorted from mouse lung and co-cultured with primary fibroblast. (A) Fibroblasts only from PBS and bleomycin treated WT and *HPS1-/-* mice. BrdU immunostaining was used to identify proliferating cells. (B) α-smooth muscle actin (α-SMA) Immunostaining was was used to detect α-SMA expression (magnification, x40). Images were representative of 4 independent experiments.
